# Supplementary material for: Family Meeting Training Curriculum: A Multimedia Approach With Real-Time Experiential Learning for Residents
Source: MedEdPORTAL. 2020 Mar 6;16:10883. doi: 10.15766/mep_2374-8265.10883 (PMC7062545; doi:10.15766/mep_2374-8265.10883)
Supplement: Supplementary file 1 — A. Communication Basics.pptx B. Family Meeting E-Learning Project folder C. ICU Resident Orientation.pptx D. Family Meeting Resources Booklet.docx E. FMBS Tool.docx F. Global Self-Efficacy Survey.docx [file mep-16-10883-s001.zip › _Educational_Summary_Report_10883.pdf]

MedEdPORTAL® The AAMC Journal of Teaching and Learning Resources

Original Publication Open Access

Family Meeting Training Curriculum: A Multimedia Approach With Real-Time Experiential Learning for Residents

Susan A. Glod, MD, MEd*, Ashley Kang, MD, Margaret Wojnar, MD, MEd

*Corresponding author: sglod@pennstatehealth.psu.edu

Abstract

Introduction: Effective communication skills are widely recognized as an important aspect of medical practice. Several tools and curricula for communications training in medicine have been proposed, with increasing attention to the need for an evidence-based curriculum for communication with families of patients in the intensive care unit (ICU). **Methods:** We developed a curriculum for internal medicine residents rotating through the medical ICU that consisted of a didactic session introducing basic and advanced communication skills, computer-based scenarios exposing participants to commonly encountered dilemmas in simulated family meetings, and experiential learning through the opportunity to identify potential communication challenges prior to facilitating actual family meetings, followed by structured peer debriefing. Seventeen residents participated in the study. **Results:** We administered the Communication Skills Attitude Scale to participants before and after participation in the curriculum, as well as a global self-efficacy survey, with some items based on the Common Ground rating instrument, at the end of the academic year. There were no significant changes in either positive or negative attitudes toward learning communication skills. Resident self-perceived efficacy in several content domains improved but did not reach statistical significance. **Discussion:** Our curriculum provided interactive preparatory training and an authentic experience for learners to develop skills in family meeting facilitation. Learners responded favorably to the curriculum. Use of the Family Meeting Behavioral Skills (FMBS) tool helped residents and educators identify and focus on specific skills related to the family meeting. Next steps include gathering and analyzing data from the FMBS tool.

Keywords
Communication, Family Meeting, Critical Care, Intensive Care Unit, End of Life, Communication Skills, Critical Care Medicine, Geriatrics, Hospice & Palliative Medicine, Hospital Medicine, Internal Medicine

Educational Objectives

By the end of this activity, learners will be able to:

1. Demonstrate positive attitudes toward the value of learning communication skills, as measured by Communication Skills Attitude Scale scores.
2. Describe the importance of the premeeting huddle and postmeeting debriefing as measured by an end-of-program-year survey.
3. Identify areas for improvement of communication skills using a global self-efficacy survey.
4. Facilitate a family meeting in real time with peer feedback using the Family Meeting Behavioral Skills tool.

Citation:
Glod SA, Kang A, Wojnar M. Family meeting training curriculum: a multimedia approach with real-time experiential learning for residents. *MedEdPORTAL.* 2020;16:10883.
https://doi.org/10.15766/mep_2374-8265.10883

Introduction

Clearly communicated information between patients and physicians allows for appropriate medical decisions and is linked to improvements in the safety, quality, and cost-effectiveness of American health care.[1] Effective communication influences patient and family satisfaction and improves medical care.[2-4] However, evidence suggests that as a profession, physicians miss key clues during discussions with families or fail to communicate basic information, such as a diagnosis, to families of patients in the intensive care unit (ICU) during encounters, sometimes more than half the time.[5-7]

A family meeting is a specific way of communicating information that can include the discussion of diagnoses, prognosis, and future care needs of the patient. This form of communication differs slightly from a patient-provider discussion, since the patient is usually too ill to participate and family members provide representation and substituted judgment on behalf of the patient.

Copyright © 2020 Glod et al. This is an open-access publication distributed under the terms of the Creative Commons Attribution license.

Historically, medical residents have learned how to facilitate family meetings through observation of their attending physicians during real-time encounters with families in the acute setting or outpatient practice. In our internal medicine residency training program, a substantial number of the family meetings in which our residents participate occur during their medical ICU (MICU) rotation.[8] When this form of instruction was used alone, members of the interdisciplinary team in our MICU felt that our residents were not always effective when facilitating meetings and proposed that additional training would be useful. We realized that formal training in communication within our residency curriculum was limited, particularly when it came to how best to communicate with families.

This discussion helped us frame some guiding principles for curriculum development. Communication skills training should provide a combination of authentic clinical experiences with feedback, along with rehearsal experiences in a safe environment to allow residents to test their skills. The training should provide opportunities for self-assessment and reflection. Finally, although acknowledging that a basic framework for communication skills is important, particularly for beginning learners, we wished to encourage deeper self-assessment and reflection during and after family interactions to promote skills development. We postulated that a curriculum that adhered to these principles would help our learners become facile, adaptable, and self-reflective in their communication, allowing them to navigate the complex communication challenges within the ICU.

Communication skills for medical trainees are teachable and measureable.[9,10] Investigators in the specialty areas of oncology, palliative medicine, and geriatric medicine have developed specific programs in communication skills for learners in their practices. These programs are designed to train either fellows or attendings in complex family meeting situations.[11-13] Other training programs have been developed specifically for family meetings that occur in the ICU.[14,15] These use a variety of educational methods including simulation and checklist-style frameworks for teaching communication skills. Many programs for ICU family meeting training are directed at the fellow level of medical experience.[16-19] However, less experienced residents with only 0-3 years of training also often spend a substantial period of time working in the ICU.

When developing our curriculum, we felt that although it was important to provide a basic framework to which residents could refer, our main focus should be to promote deeper learning by embedding the curriculum in authentic clinical experiences. Other educators have emphasized and encouraged direct performance of family meetings with feedback, arguing that using other methods, such as the interview of standardized patients, is like "predicting baseball teams' performance by watching exhibition games."[20] Our goal was to incorporate specific advanced skills into everyday workflow, with emphasis on direct observation and feedback, to highlight proper communication performance and feedback for the rounding team,[10,20] as well as potentially influencing the existing culture around this topic in a positive way. We wanted to move beyond specific models of skills training, such as giving bad news, or acronym-based models of training, so that residents could instead practice applying specific skills (e.g., eliciting perspectives, managing uncertainty, sharing information) in a variety of clinical scenarios.

We sought out a tool that could be used in real clinical interactions to allow for self-assessment and peer observation. Others have developed and studied a large number of tools for direct assessment of general communication competency that range in their psychometric properties.[21] Two tools, the Family Meeting Behavioral Skills (FMBS) tool and the Family Meeting Communication Assessment Tool, have been developed and used in two large studies looking at skill acquisition of communication skills by fellows.[13,18] The FMBS instrument was studied and validated, with internal consistency noted across the checklist of items on the instrument (α reliability = .855), as well as strong correlation between the number of skills performed and the level of skill ($r = .827$, $p < .01$) and interjudge reliability of .571.[22] The FMBS was scored by two psycho-oncology postdoctoral fellows who measured performance of pulmonary and critical care fellows from taped interviews, with the ICU used as the area of study. The FMBS includes a self-reflective question on potential challenges that the learner is expected to encounter, a direct observation checklist, and a structured debriefing section after the session is completed. We chose to use this tool for our study because of its focus on self-reflection and structured debriefing.

Although more investigators are studying ways to improve general communication skills and family meeting skills, many gaps remain. The complexity of family meetings makes defining effective communication in a range of scenarios challenging. Another gap in the literature is the use of learners' self-perception of self-confidence as an assessment of the program training them. Self-perceived improvement is noted by programs and seems to be a consistent measure in studies; however, self-perception and self-rating are unreliable by themselves.[23] Data on the use of direct observation during actual clinical encounters

Copyright © 2020 Glod et al. This is an open-access publication distributed under the terms of the Creative Commons Attribution license.

are limited,[24] and it is not clear how reliable the use of direct observation for feedback by members of an interprofessional group would be.

This curriculum builds on prior work in our MICU,[8] during which we piloted a different peer observation tool, the Common Ground rating instrument,[25] to be used by a peer during family meeting facilitation in order to provide feedback to the facilitating resident. Our educational objectives for the initial curriculum were to allow trainees to (1) apply a framework of specific communication skills to family conference facilitation, (2) use the modified Common Ground Assessment Instrument (mCGAI) to rate the quality of family conference facilitation, and (3) provide formative feedback on family conference facilitation to a peer using the mCGAI. We surveyed residents to ascertain how often and to what extent they facilitated family meetings, as well as their confidence in doing so. The results that we gathered during the initial curriculum demonstrated that our residents infrequently facilitated family meetings but nonetheless expressed high levels of confidence in their ability to do so. We also received feedback from members of the interprofessional ICU team that some residents were still struggling during family meeting facilitation. Our goal was to allow residents the opportunity to develop communication skills around common ICU communication scenarios in a low-stakes computer-based environment while simultaneously interacting with real families during actual clinical experiences in the ICU. Therefore, as part of the new curriculum, we have added computer-based simulation and self-reflection to support better calibration and opportunities for skill development prior to the facilitation of actual meetings. There are four parts to the program. The program's time line is as follows:

- Part 1: introductory interactive session—once, at the beginning of the academic year, for all residents.
- Part 2: interactive computer-based modules—once for each resident, asynchronously, online prior to starting the scheduled MICU rotation.
- Part 3: MICU introduction session—once a month, during orientation for residents starting the MICU rotation.
- Part 4: family meeting facilitation with self-reflection, peer feedback, and self-assessment—ad lib during the resident's MICU rotation whenever a family meeting occurs.

There are several other publications available in *MedEdPORTAL* that address challenging communication scenarios. A full review of these is outside the scope of this report; however, examples include the following. Lamba, Bryczkowski, Holland, Nagurka, and Mosenthal[26] used teaching objective structured clinical examinations (OSCEs) with review by peers to provide feedback to trauma surgery residents when communicating difficult news. Cannone, Atlas, Fornari, Barilla-LaBarca, and Hoffman[27] used pre- and postvideo OSCEs with an intervening communication curriculum to prepare oncology residents and fellows to deliver difficult news. Reed and Sharma[28] used brief OSCEs with real-time video broadcast to allow learners who were not participating in the examinations to provide peer feedback afterward. Our work is unique in that it incorporates asynchronous interactive flat-screen simulation to develop core concepts in communication, with the addition of peer feedback during actual family meetings.

Methods

Our goal was to address the problem of insufficient family meeting communication skills training by developing a curriculum for graduate medical trainees that provided just-in-time learning around facilitation of family meetings during the MICU rotation. We wanted to provide residents with a review of basic communication skills, as well as a framework that they could use during real-time family meetings during their ICU rotation, with opportunities for peer feedback. Prior to facilitating actual family meetings, we wanted residents to have a safe opportunity for practicing skills via a series of computer-based simulations. It was important to us to minimize curricular demands on the residents during their busy ICU month by embedding the training directly in their clinical rotation as much as possible. Considering that the residents and ICU staff had expressed interest in improving communication with families in the ICU, it was not difficult to obtain buy-in from the residents, program director, and ICU interprofessional teams, particularly since the structure of the curriculum minimized time away from patient care.

We administered the program to internal medicine residents during their MICU rotation. The time line for the curriculum extended over 1 year and included 34 residents in total, with groups of learners rotating in and out as they engaged in ICU rotations.

We presented the first session, which was an interactive introduction reviewing basic and complex communication skills and how they apply to family meetings in the ICU, during a 1-hour time slot in the general internal medicine residency training program core curriculum sessions at the beginning of the academic year (Appendix A). The need for this session was multifactorial: Many residents had had limited exposure to the basics of communication. Identifying the elements of basic and intermediate skills was needed prior to the more complex

Copyright © 2020 Glod et al. This is an open-access publication distributed under the terms of the Creative Commons Attribution license.

family meeting skills. The introductory session was one means of assuring that all residents had a baseline exposure to these skills. Highlighting communication skills brought attention to them as something the residents needed to learn. We felt that the emphasis on self-assessment of skill development of family meeting facilitation was important to introduce before the ICU rotation. During this session, we also provided the residents with instructions (described below) on how to access the scenarios and asked them to complete the scenarios prior to starting their MICU rotation.

The second curricular component was the interactive computer-based component (Appendix B). To operate the web-based component in Appendix B, follow these steps:

1. Download and open the zip file.
2. Remove the content folder from the zip file and place on your computer's desktop.
3. Open the folder.
4. Access the story.html file by right-clicking and opening with your browser.

This component allowed for common, unusual, challenging, and/or infrequently encountered scenarios to be shared with the learner. We asked the residents to work through the problems posed by the scenarios at their own pace. The program then allowed them to select and observe the effects of their answers. This provided a protected or safe method of learning, where decisions were trialed without harm to the patient or family. The program utilized four cases that we developed based on a needs assessment from our residents on the types of family meetings they found challenging. The cases included a family member misunderstanding the severity of illness, an angry family member, family members who had different views on the patient's wishes, and a family's unreasonable expectations under the current medical conditions. The cases were developed by expert faculty familiar with family meetings, who were filmed interacting with simulated family members. Each case began with a video clip, followed by multiple-choice questions that asked the learner to choose the best response to a family member's statement or question. Depending on the selected answer, the interactive media played a follow-up video conversation. If the learner did not like how the scenario was going, he or she could go back and choose a different response until satisfied with how the scenario ended. By utilizing this method, the residents were exposed to challenging scenarios prior to live exposure, with the goal that when encountering the real-life situation, the learners would have already worked through a similar event and have a greater chance of being successful. Although the number of actors in the video scenarios was small, often resulting in only the physician and family member alone on tape during the encounter, we stressed to the residents during our other sessions that they should envision the actors as members of a full interdisciplinary team, the rest of which was behind the camera.

The third component of the curriculum was delivered at the start of the MICU rotation. This consisted of a 30-minute interactive session delivered during the first several days of the MICU rotation (Appendix C). This session was co-led by a member of the ICU interdisciplinary team and a physician. During the session, basic communication skills were reviewed, and logistical information about the structure and function of family meetings was discussed. This session provided logistical information about how our ICU scheduled and facilitated family meetings. Residents were also provided with the family meeting resource booklet[29] (Appendix D), which we authored for internal use to provide logistical information, as well as tips and tricks for facilitation of family meetings. Finally, this session introduced the last portion of the curriculum: peer observation and feedback on actual family meeting facilitation.

During this fourth portion of the curriculum, the residents were asked to participate in actual family meetings using the family meeting blueprint included in the resource booklet along with the FMBS tool (Appendix E). Opportunities to facilitate existed throughout the 4 weeks of ICU assigned to each resident. The family meeting was viewed as an interactive session whereby the learner started by observing a family meeting with the goal of eventually taking the lead in running one. A care coordinator and social worker were part of the team and usually arranged and guided the family meetings, depending on the residents' role in the patient's care. Individuals trained in running family meetings and supporting learners were involved in the actual meetings. These individuals included a member of the palliative care service, a social worker, a nurse care coordinator, and an ICU attending or ICU fellow. The meetings followed the blueprint outlined in the resource booklet provided at the didactic lecture. As per the structure of the family meeting blueprint, a premeeting was done to discuss the goals of that particular meeting. Responsibilities for the meeting were assigned—that is, the person who was to lead the meeting was designated. The resident then facilitated the meeting with assistance, if needed, from more senior team members. After the meeting concluded, there was a debriefing session with the members of the team. Any new plans related to the goals of the meeting were discussed and then outlined in a family meeting note by a designated member of the team. Feedback to the resident

Copyright © 2020 Glod et al. This is an open-access publication distributed under the terms of the Creative Commons Attribution license.

was provided using the FMBS tool by other members of the interdisciplinary team who were present at the meeting.

We administered the Communication Skills Attitude Scale (CSAS)[30] to participants before and after their completion of the interactive online cases. The CSAS was a 26-item validated tool measuring participants' attitudes toward learning communication skills. It included both positive attitude items (e.g., "Developing my communication skills is just as important as developing my knowledge of medicine") and negative attitude items (e.g., "I do not need good communication skills to be a doctor"). The CSAS is not a required component of the published curriculum. Additionally, we used the FMBS tool both as a means of self-assessment and as a way of providing formative feedback to participants after they facilitated actual family meetings.

At the end of the academic year, we also administered a global self-assessment survey (Appendix F), with some items based on of the Common Ground instrument,[25] to residents who had participated in the curriculum. The survey was used for data collection but is not a required part of the published curriculum. In the survey, we asked residents to describe the frequency of family meeting facilitation during their MICU rotation, self-assess their use of favorable communication behaviors (e.g., addressing feelings and emotions, listening and responding to cues from family members, and checking for understanding and agreement) during meetings, and rate the importance of components of the meeting, such as the premeeting huddle. We also asked residents to rate their overall effectiveness in family meeting facilitation and to list challenges to receiving feedback on their facilitation skills. We compared these data to data from identical surveys that we had administered 3 years earlier, prior to full implementation of the curriculum.

Results

There were 34 residents in our program. We did not track how many of them were present for the lecture-based portions of the curriculum. We administered the computer-based and peer observation portions of the curriculum over 6 months to 24 residents. We administered the global self-efficacy survey to all of them.

Frequency of Facilitation

The global self-efficacy survey included items that asked participants how often they had facilitated family conferences in the past year, as well as items related to global self-assessment of their communication skills. The self-efficacy survey was given to 34 residents, 19 of whom completed it (56%). Of those, more than half (58%) stated that they had served as a primary facilitator for three or fewer family conferences over the past year of training. Thirty-four percent indicated that they had served as primary facilitator for four to six family meetings over the past year, and 26% indicated that they had facilitated seven or more meetings over the past year. This represented a trend toward more primary facilitation of conferences within our residency program as compared to self-reported family conference facilitation when we administered the same survey 3 years earlier, at which time more than 90% of respondents ($n = 32$) indicated that they had facilitated six or fewer family conferences in the past year.

Self-Assessment Data

Global self-assessment items from the global self-efficacy survey were analyzed using the Wilcoxon rank sum test. Results are described in the Table. Results labeled *Pre* are from the survey administered to the resident class in 2015, prior to the full rollout of this curriculum. Results labeled *Post* are from the survey administered to our current class of residents after participation in the curriculum. Although ratings of some skills increased, no changes in skill rating reached statistical significance.

CSAS Scores

We administered the curriculum over 6 months to a total of 24 internal medicine residents. Eight of these participants completed the CSAS both pre- and postcurriculum, and we used the Wilcoxon signed rank test to look for significant changes in the Positive Attitude Scale or Negative Attitude Scale. CSAS results did not show any significant changes in either positive (Pre = 4.03, Post = 3.97, $p = .688$) or negative (Pre = 2.21, Post = 2.10, $p = .406$) attitudes toward learning communication skills. We did not measure the number of completed FMBS tools, as these were provided directly to the residents for formative feedback. We did not formally survey residents about perceived efficacy of the curriculum.

Qualitative Survey Themes

Within the global self-efficacy survey, we asked three open-ended questions: (1) List three things that you have learned about family meetings during the rotation. (2) What family meeting facilitation skills have you improved upon during your MICU rotation? (3) If feedback is important to you, can you identify impediments to getting feedback for how you facilitate family meetings?

The following themes were identified.

Preparedness: Residents recognized the importance of preparing both themselves and the team as a whole for a

Copyright © 2020 Glod et al. This is an open-access publication distributed under the terms of the Creative Commons Attribution license.

Table. Resident Self-Assessment Survey Results

Question[a] and Group[b]	*M*	*SD*	95% Confidence Interval for *M*	Quartile Range	*p*[c]
When I facilitate a family conference, I effectively develop rapport with families.					
Pre	3.9	0.54	3.7-4.2	0.00	.061
Post	4.3	0.47	4.0-4.6	1.00	
When I facilitate a family conference, I ask open-ended questions effectively.					
Pre	4.1	0.32	4.1-4.3	0.00	.833
Post	4.1	0.47	3.8-4.4	0.00	
When I facilitate a family conference, I elicit all of the family's agenda items.					
Pre	3.7	0.67	3.4-4.1	1.00	.162
Post	4.1	0.62	3.7-4.4	0.00	
When I facilitate a family conference, I listen for and respond to cues to the family's ideas, concerns, and expectations.					
Pre	4.1	0.90	3.7-4.6	1.00	.523
Post	4.4	0.63	4.0-4.7	1.00	
When I facilitate a family conference, I address feelings and emotions with the family.					
Pre	4.1	0.76	3.7-4.5	1.00	.540
Post	4.3	0.73	3.9-4.7	1.00	
When I facilitate a family conference, I check for understanding and agreement from the family when deciding on a plan of care.					
Pre	4.4	0.70	4.1-4.8	1.00	.749
Post	4.4	0.51	4.1-4.7	1.00	
My overall rating of family conference facilitation skills is:					
Pre	3.8	0.62	3.5-4.1	1.00	.290
Post	4.1	0.62	3.7-4.4	0.00	

[a]Rated 1-5.
[b]The Pre group was the resident class in 2015, prior to the full rollout of this curriculum. The Post group is our current class of residents after participation in the curriculum.
[c]Wilcoxon rank sum test.

family meeting before the start of the meeting (premeeting needs):

- "It is crucial to be prepared with the patient's course, diagnosis, and treatment."
- "Try to schedule in advance and plan strategy with entire care team involved."

Silence: Respondents noted the importance of silence in allowing families to process and retain information, as well as to process emotions (meeting skills):

- "Leave space for patient/family to talk."
- "Silence is okay."

It's about the family, not you: The residents identified the need to prioritize the needs of a family over all other concerns and to extract their own personal and professional goals from the conversations (meeting skills):

- "Be forthcoming about the patient's condition and prognosis. Don't have an agenda before entering the meeting."
- "Listening to the patient/family/next of kin and identifying their goals."
- "Don't take the family's decision personally."

Time as a barrier to feedback: Almost all respondents who identified a barrier to receiving feedback listed time as the major challenge (debriefing):

- "Time limitations and other duties for all participants."

Discussion

Family meetings involve complex communication skills, the development of which relies heavily on self-assessment, self-reflection, and rehearsal. We believe that our curriculum filled a gap in the literature by combining multiple modalities of learning, from basic skills training in a lecture format to a safe, computer-based simulation that allowed learners to choose different responses, observe their effects, and recalibrate. Our curriculum also engaged our learners in authentic patient care experiences, allowing them to self-assess, receive feedback, and then self-reflect on their performance.

The use of the CSAS tool allowed us to gauge the attitudes of our learners prior to beginning the curriculum. The use of pre/post self-assessment tools provided the opportunity for learners to reflect on their own skills and areas for improvement. We believed that it was important to then review foundational communication skills with all residents prior to discussions of more complex skills. This base was part of the scaffolding of

Copyright © 2020 Glod et al. This is an open-access publication distributed under the terms of the Creative Commons Attribution license.

information used to construct the curriculum utilizing situated learning theory.[31] The computer-based simulation, although self-guided, provided additional layers and testing of skills as a supportive experience. This type of safe and reassuring feedback was a form of scaffolding seen in situated learning.[32] The second didactic lecture reinforced information before the learner actually performed the family meeting. Additional scaffolding and focus on communication as a skill were incorporated into the actual family meeting with guidance from the FMBS tool. Unlike the CSAS tool, which measured attitudes toward learning communication skills, the FMBS tool measured actual behaviors associated with good communication. The interdisciplinary team member who attended the actual family meeting acted as a facilitator, allowing the learner to actively participate within his or her ability, but was present to provide support as needed. The curriculum held a framework of information building on basic information, providing opportunities for rehearsal and self-assessment, and then guiding the learner through the actual experience with the help of experienced interdisciplinary staff.

Allowing residents to lead family meetings provided them with an authentic experience. This emphasis on having the resident lead family meetings was based on feedback from our residents[8] and from the literature, in which learners expressed the need to perform these meetings to test what they had learned from other experiences.[33-35] This emphasis is consistent with situated learning theory, where the learners are placed in authentic situations and supported by those around them until they are successful in managing the situations by themselves.[33,36] The format of our program followed recommendations by McLellan[36] by providing opportunities for multiple practices with authentic scenarios, collaboration, and reflection on practice.

Any program design must consider the balance of many factors, including the use of time. We designed our curriculum to minimize time in recognition of what the resident already had to do in a day for his or her patients. The initial lecture for the residents took place during a time already designated for resident educational sessions. The ICU rotation didactic was given during each block's orientation session for the residents over lunch. The computer-based simulation was chosen as a method over scheduled simulated actor exposure in real time so that the residents could access the simulation whenever there was time available. The actual family meetings occured as needed but usually were in the afternoons or evenings, when family members were able to attend. The timing of these sessions was part of the normal workday for residents.

One limitation of our program was the purposeful choice of not using video recordings of residents interacting with simulated actors, a method used in other programs found in the literature.[16,18,37] Although using the video-recording method provides a safe learning environment, allows for practice and feedback, and is a powerful tool to demonstrate a learner's behavior under different circumstances, there is no evidence to show that this method versus live participation with direct observational feedback provides an educational advantage or improves patient and family satisfaction. This concept of direct comparison of methods of simulated communication and feedback compared to live simulation training and feedback is ripe for future research.

Another limitation of the program was the small sample size. Our institution hosts a midsized cohort of residents, only a few of whom are rotating in the ICU at any given time. Because of this, we did not directly assess the impact of our curriculum on patient-related outcomes, including patient and family satisfaction, ICU or hospital length of stay, or direct assessment of patient and/or family understanding of the disease process or prognosis. What we did assess, using a self-reflection survey and qualitative analysis, was a trend (albeit not robust) toward more primary facilitation, building rapport with families and eliciting a family's agenda. This was supported by the comments from the qualitative analysis, where the residents noted the importance of "space for patient/family to talk" and the use of silence as a tool when getting to the family's agenda. Other comments supported the residents' awareness of the meeting being about the patient and the family. Although our qualitative analysis did not reach saturation, in acknowledging the importance of preparedness, leaving one's own agenda out of the meeting, and using specific tools like silence, the comments that were gathered suggest our key objectives were supported by the curriculum. It is possible that the small sample size contributed to the lack of significant statistical differences between the pre- and post-CSAS scores and self-assessment scores.

We also note the fact that this curriculum required substantial faculty support to be maintained and should not be viewed as a self-sustaining curriculum once implemented. Buy-in regarding the importance of these skills was needed from residents, faculty, and the entire multidisciplinary ICU team for optimal success. Building on the informal favorable feedback utilizing the FMBS form for actual family meetings would be a natural next step to focus on identifying and obtaining specific skill acquisition.

Copyright © 2020 Glod et al. This is an open-access publication distributed under the terms of the Creative Commons Attribution license.

Appendices

A. Communication Basics.pptx

B. Family Meeting E-Learning Project folder

C. ICU Resident Orientation.pptx

D. Family Meeting Resources Booklet.docx

E. FMBS Tool.docx

F. Global Self-Efficacy Survey.docx

All appendices are peer reviewed as integral parts of the Original Publication.

Susan A. Glod, MD, MEd: Associate Professor, Department of Medicine, Penn State College of Medicine; Medicine Clerkship Director, Penn State College of Medicine

Ashley Kang, MD: Resident, Internal Medicine Residency Program, Montefiore Medical Center

Margaret Wojnar, MD, MEd: Professor, Department of Medicine, Penn State College of Medicine; Pulmonary/Critical Care Fellowship Director, Penn State College of Medicine

---

Acknowledgments
The authors would like to acknowledge Erik Lehman for assistance in statistical analysis.

Disclosures
None to report.

Funding/Support
None to report.

Informed Consent
All identifiable persons in this resource have granted their permission.

Ethical Approval
The Penn State Human Subjects Protection Office approved this study.

References

1. Epstein RM, Fiscella K, Lesser CS, Stange KC. Why the nation needs a policy push on patient-centered health care. *Health Aff (Millwood).* 2010;29(8):1489-1495. https://doi.org/10.1377/hlthaff.2009.0888
2. Hinkle LJ, Bosslet GT, Torke AM. Factors associated with family satisfaction with end-of-life care in the ICU. *Chest.* 2015;147(1):82-93. https://doi.org/10.1378/chest.14-1098
3. Hwang DY, Yagoda D, Perrey HM, et al. Assessment of satisfaction with care among family members of survivors in a neuroscience intensive care unit. *J Neurosci Nurs.* 2014;46(2):106-116. https://doi.org/10.1097/JNN.0000000000000038
4. Curtis JR, Engelberg RA, Wenrich MD, et al. Studying communication about end-of-life care during the ICU family conference: development of a framework. *J Crit Care.* 2002;17(3):147-160. https://doi.org/10.1053/jcrc.2002.35929
5. Curtis JR, Engelberg RA, Wenrich MD, Shannon SE, Treece PD, Rubenfeld GD. Missed opportunities during family conferences about end-of-life care in the intensive care unit. *Am J Respir Crit Care Med.* 2005;171(8):844-849. https://doi.org/10.1164/rccm.200409-1267OC
6. McDonagh JR, Elliott TB, Engelberg RA, et al. Family satisfaction with family conferences about end-of-life care in the intensive care unit: increased proportion of family speech is associated with increased satisfaction. *Crit Care Med.* 2004; 32(7):1484-1488. https://doi.org/10.1097/01.CCM.0000127262.16690.65
7. Azoulay E, Chevret S, Leleu G, et al. Half the families of intensive care unit patients experience inadequate communication with physicians. *Crit Care Med.* 2000;28(8):3044-3049. https://doi.org/10.1097/00003246-200008000-00061
8. Glod S, Wojnar M, Kreher M, Smith ML. A curriculum to enhance resident communication skills during family conferences in the intensive care unit. *MedEdPORTAL.* 2015;11:10305. https://doi.org/10.15766/mep_2374-8265.10305
9. Brown RF, Bylund CL. Communication skills training: describing a new conceptual model. *Acad Med.* 2008;83(1):37-44. https://doi.org/10.1097/ACM.0b013e31815c631e
10. Rider EA, Hinrichs MM, Lown BA. A model for communication skills assessment across the undergraduate curriculum. *Med Teach.* 2006;28(5):e127-e134. https://doi.org/10.1080/01421590600726540
11. Back AL, Arnold RM, Baile WF, et al. Efficacy of communication skills training for giving bad news and discussing transitions to palliative care. *Arch Intern Med.* 2007;167(5):453-460. https://doi.org/10.1001/archinte.167.5.453
12. Bylund CL, Brown RF, Bialer PA, Levin TT, Lubrano di Ciccone B, Kissane DW. Developing and implementing an advanced communication training program in oncology at a comprehensive cancer center. *J Cancer Educ.* 2011;26(4):604-611. https://doi.org/10.1007/s13187-011-0226-y
13. Gelfman LP, Lindenberger E, Fernandez H, et al. The effectiveness of the Geritalk communication skills course: a real-time assessment of skill acquisition and deliberate practice. *J Pain Symptom Manage.* 2014;48(4):738-744. https://doi.org/10.1016/j.jpainsymman.2013.12.231
14. Sullivan AM, Rock LK, Gadmer NM, Norwich DE, Schwartzstein RM. The impact of resident training on communication with families in the intensive care unit: resident and family outcomes. *Ann Am Thorac Soc.* 2016;13(4):512-521. https://doi.org/10.1513/AnnalsATS.201508-495OC

Copyright © 2020 Glod et al. This is an open-access publication distributed under the terms of the Creative Commons Attribution license.

15. Curtis JR, Back AL, Ford DW, et al. Effect of communication skills training for residents and nurse practitioners on quality of communication with patients with serious illness: a randomized trial. *JAMA*. 2013;310(21):2271-2281. https://doi.org/10.1001/jama.2013.282081

16. Arnold RM, Back AL, Barnato AE, et al. The Critical Care Communication project: improving fellows' communication skills. *J Crit Care*. 2015;30(2):250-254. https://doi.org/10.1016/j.jcrc.2014.11.016

17. Berman EM, Heru AM, Grunebaum H, Rolland J, Wood B, Bruty H; and Group for the Advancement of Psychiatry Committee on the Family. Family skills for general psychiatry residents: meeting ACGME core competency requirements. *Acad Psychiatry*. 2006;30(1):69-78. https://doi.org/10.1176/appi.ap.30.1.69

18. McCallister JW, Gustin JL, Wells-Di Gregorio S, Way DP, Mastronarde JG. Communication skills training curriculum for pulmonary and critical care fellows. *Ann Am Thorac Soc*. 2015;12(4):520-525. https://doi.org/10.1513/AnnalsATS.201501-039OC

19. Montagnini M, Varkey B, Duthie E Jr. Palliative care education integrated into a geriatrics rotation for resident physicians. *J Palliat Med*. 2004;7(5):652-659. https://doi.org/10.1089/jpm.2004.7.652

20. Henry SG, Holmboe ES, Frankel RM. Evidence-based competencies for improving communication skills in graduate medical education: a review with suggestions for implementation. *Med Teach*. 2013;35(5):395-403. https://doi.org/10.3109/0142159X.2013.769677

21. Schirmer JM, Mauksch L, Lang F, et al. Assessing communication competence: a review of current tools. *Fam Med*. 2005;37(3):184-192.

22. Gustin J, McCallister J, Way D, Frier K, Wells S. Validation of a family meeting behavioral skills (FMBS) instrument. Presented at: 16th Ottawa Conference on the Assessment of Competence in Medicine and the Healthcare Professions; April 25-29, 2014; Ottawa, ON, Canada. https://www.researchgate.net/publication/269709837_Validation_of_a_family_meeting_behavior_skills_(FMBS)_instrument

23. Dickson RP, Engelberg RA, Back AL, Ford DW, Curtis JR. Internal medicine trainee self-assessments of end-of-life communication skills do not predict assessments of patients, families, or clinician-evaluators. *J Palliat Med*. 2012;15(4):418-426. https://doi.org/10.1089/jpm.2011.0386

24. Gustin JL, Way DP, Wells-Di Gregorio S, McCallister JW. Validation of the Family Meeting Behavioral Skills Checklist: an instrument to assess fellows' communication skills. *Ann Am Thorac Soc*. 2016;13(8):1388-1393. https://doi.org/10.1513/AnnalsATS.201601-021OC

25. Kreher M, Wojnar M, Glod S. *Family Meeting Reference Guide*. Hershey, PA: Pennsylvania State College of Medicine and Milton S. Hershey Medical Center; 2015.

26. Lamba S, Bryczkowski S, Holland B, Nagurka R, Mosenthal A. A teaching OSCE to assess trauma resident skills on how to hold difficult conversations with family of critically injured patients. *MedEdPORTAL*. 2016;12:10398. https://doi.org/10.15766/mep_2374-8265.10398

27. Cannone D, Atlas M, Fornari A, Barilla-LaBarca M-L, Hoffman M. Delivering challenging news: an illness-trajectory communication curriculum for multispecialty oncology residents and fellows. *MedEdPORTAL*. 2019;15:10819. https://doi.org/10.15766/mep_2374-8265.10819

28. Reed DJ-W, Sharma J. Delivering difficult news and improving family communication: simulation for neonatal-perinatal fellows. *MedEdPORTAL*. 2016;12:10467. https://doi.org/10.15766/mep_2374-8265.10467

29. Rees C, Sheard C, Davies S. The development of a scale to measure medical students' attitudes towards communication skills learning: the Communication Skills Attitude Scale (CSAS). *Med Educ*. 2002;36(2):141-147. https://doi.org/10.1046/j.1365-2923.2002.01072.x

30. Lang F, McCord R, Harvill L, Anderson DS. Communication assessment using the Common Ground instrument: psychometric properties. *Fam Med*. 2004;36(3):189-198.

31. Lave J, Wenger E. *Situated Learning: Legitimate Peripheral Participation*. New York, NY: Cambridge University Press; 1991.

32. Collins A. Cognitive apprenticeship. In: Sawyer RK, ed. *The Cambridge Handbook of the Learning Sciences*. New York, NY: Cambridge University Press; 2006:47-60.

33. van den Eertwegh V, van Dalen J, van Dulmen S, van der Vleuten C, Scherpbier A. Residents' perceived barriers to communication skills learning: comparing two medical working contexts in postgraduate training. *Patient Educ Couns*. 2014;95(1):91-97. https://doi.org/10.1016/j.pec.2014.01.002

34. van den Eertwegh V, van der Vleuten C, Stalmeijer R, van Dalen J, Scherpbier A, van Dulmen S. Exploring residents' communication learning process in the workplace: a five-phase model. *PLoS One*. 2015;10(5):e0125958. https://doi.org/10.1371/journal.pone.0125958

35. Collins A. *Design Issues for Learning Environments*. New York, NY: Center for Technology in Education; 1993.

36. McLellan H, ed. *Situated Learning Perspectives*. Englewood Cliffs, NJ: Educational Technology Publications; 1996.

37. Hope AA, Hsieh SJ, Howes JM, et al. Let's talk critical: development and evaluation of a communication skills training program for critical care fellows. *Ann Am Thorac Soc*. 2015;12(4):505-511. https://doi.org/10.1513/AnnalsATS.201501-040OC

Received: December 6, 2018
Accepted: October 14, 2019
Published: March 6, 2020

Copyright © 2020 Glod et al. This is an open-access publication distributed under the terms of the Creative Commons Attribution license.
