# Supplementary material for: Family Meeting Training Curriculum: A Multimedia Approach With Real-Time Experiential Learning for Residents
Source: MedEdPORTAL. 2020 Mar 6;16:10883. doi: 10.15766/mep_2374-8265.10883 (PMC7062545; doi:10.15766/mep_2374-8265.10883)
Supplement: Supplementary file 1 — A. Communication Basics.pptx B. Family Meeting E-Learning Project folder C. ICU Resident Orientation.pptx D. Family Meeting Resources Booklet.docx E. FMBS Tool.docx F. Global Self-Efficacy Survey.docx [file mep-16-10883-s001.zip › B. Family Meeting E-Learning Project folder/story.html]

Family Meeting E-Learning Project 


|  |
| --- |
| This course cannot be played.  Learn More |
