# Supplementary material for: Family Meeting Training Curriculum: A Multimedia Approach With Real-Time Experiential Learning for Residents
Source: MedEdPORTAL. 2020 Mar 6;16:10883. doi: 10.15766/mep_2374-8265.10883 (PMC7062545; doi:10.15766/mep_2374-8265.10883)
Supplement: Supplementary file 1 — A. Communication Basics.pptx B. Family Meeting E-Learning Project folder C. ICU Resident Orientation.pptx D. Family Meeting Resources Booklet.docx E. FMBS Tool.docx F. Global Self-Efficacy Survey.docx [file mep-16-10883-s001.zip › C. ICU Resident Orientation.pptx]

## Slide 1
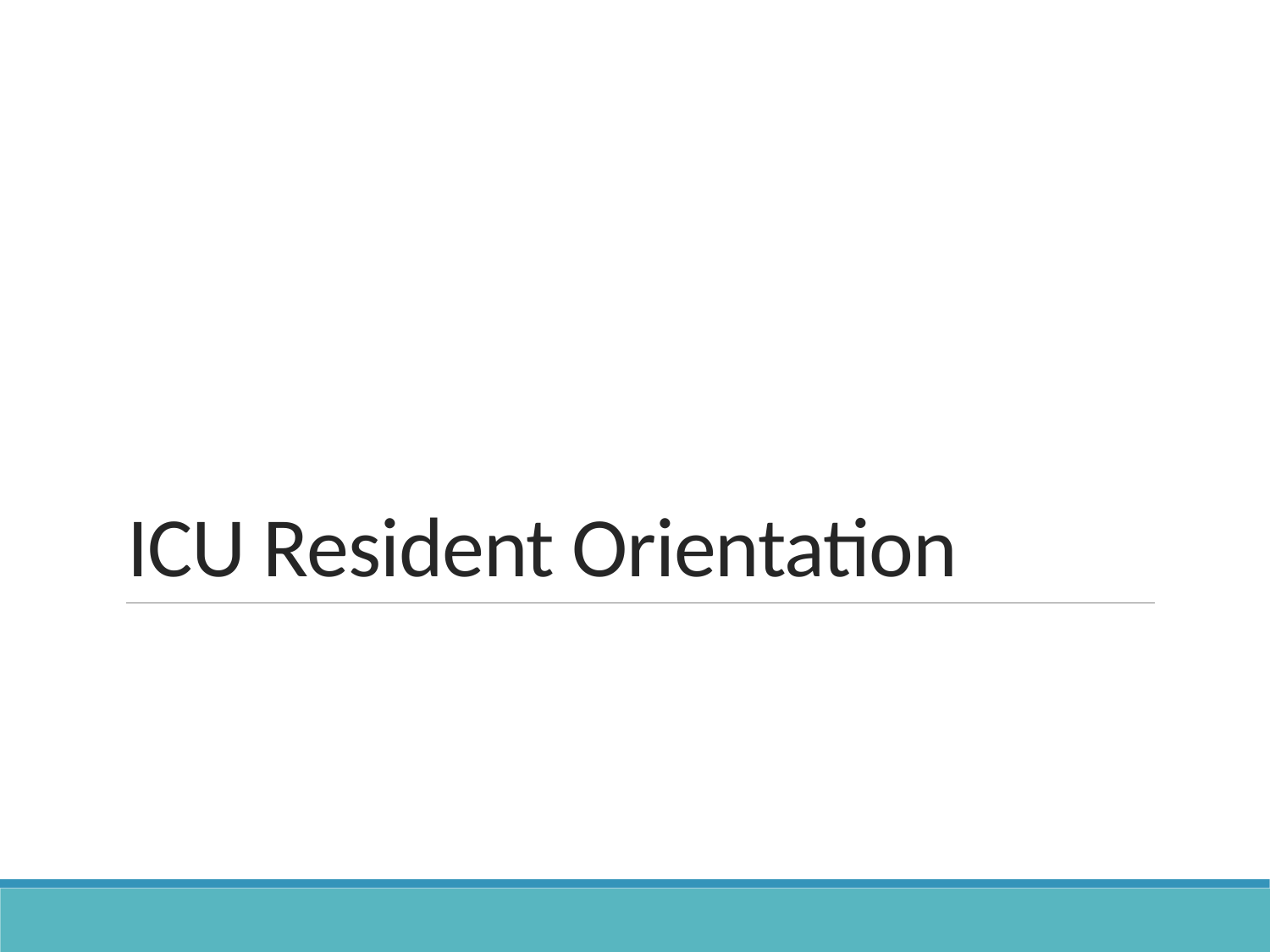

# ICU Resident Orientation

## Slide 2
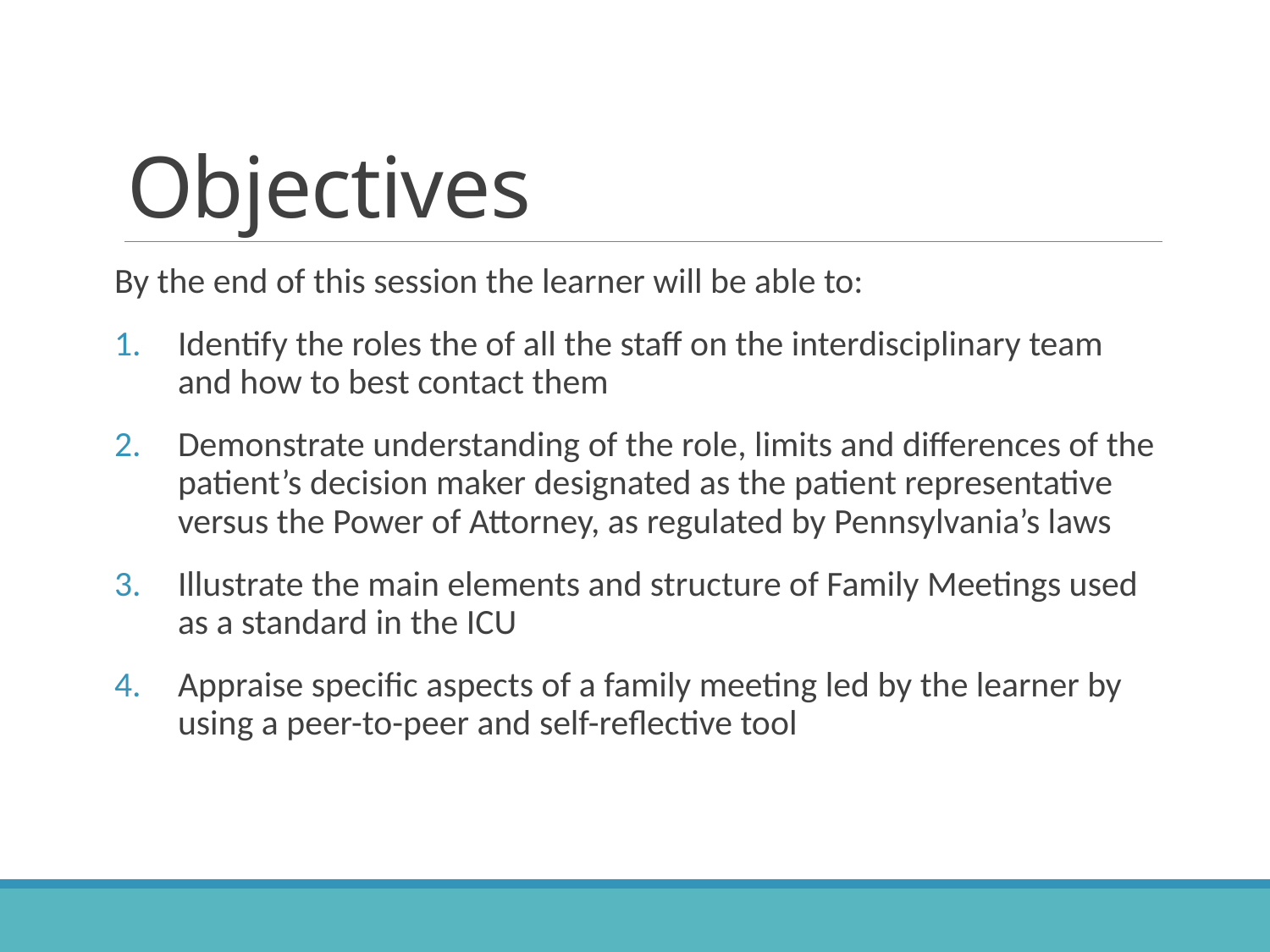

# Objectives
By the end of this session the learner will be able to:
Identify the roles the of all the staff on the interdisciplinary team and how to best contact them
Demonstrate understanding of the role, limits and differences of the patient’s decision maker designated as the patient representative versus the Power of Attorney, as regulated by Pennsylvania’s laws
Illustrate the main elements and structure of Family Meetings used as a standard in the ICU
Appraise specific aspects of a family meeting led by the learner by using a peer-to-peer and self-reflective tool

## Slide 3
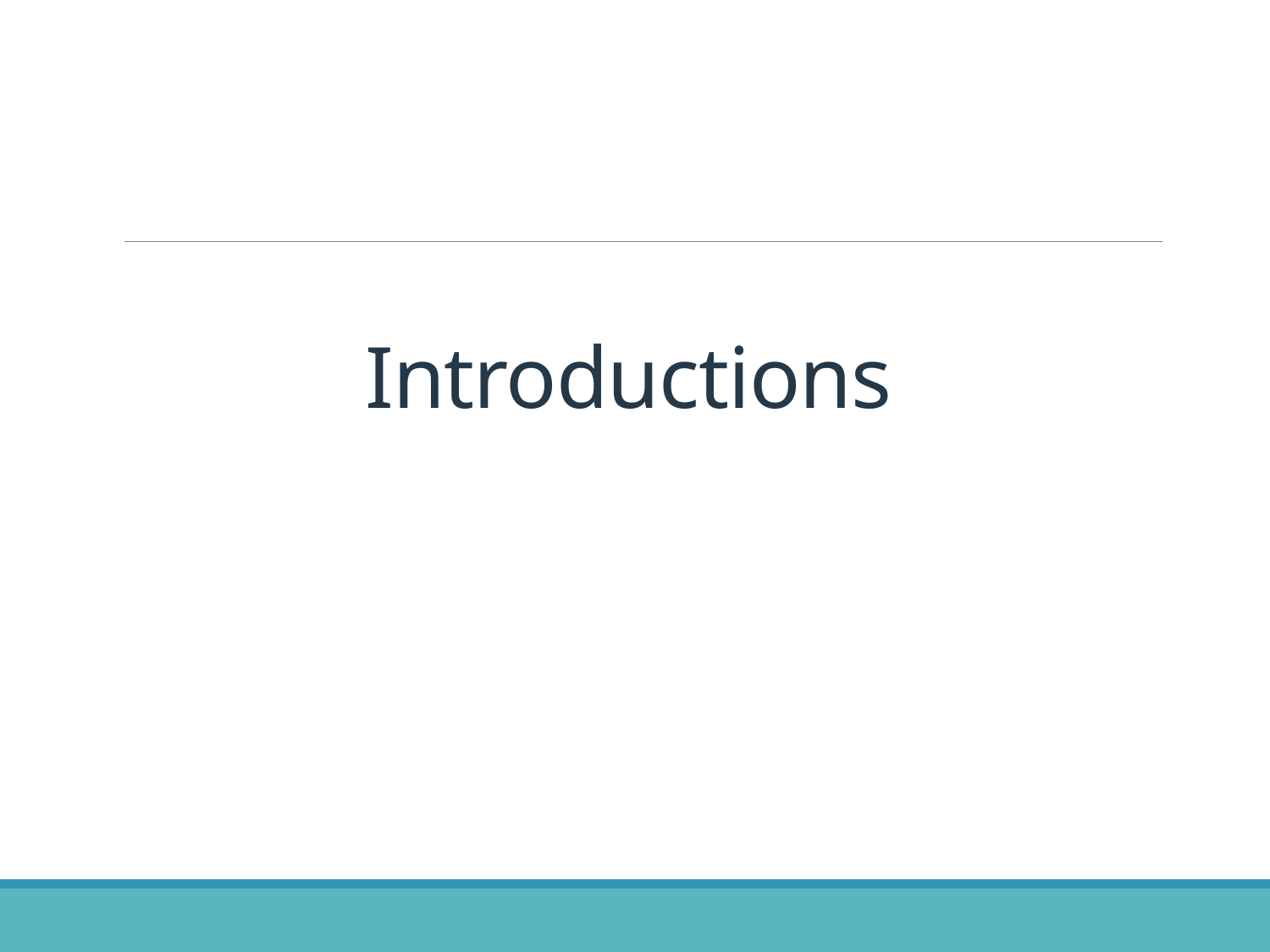

#
Introductions

## Slide 4
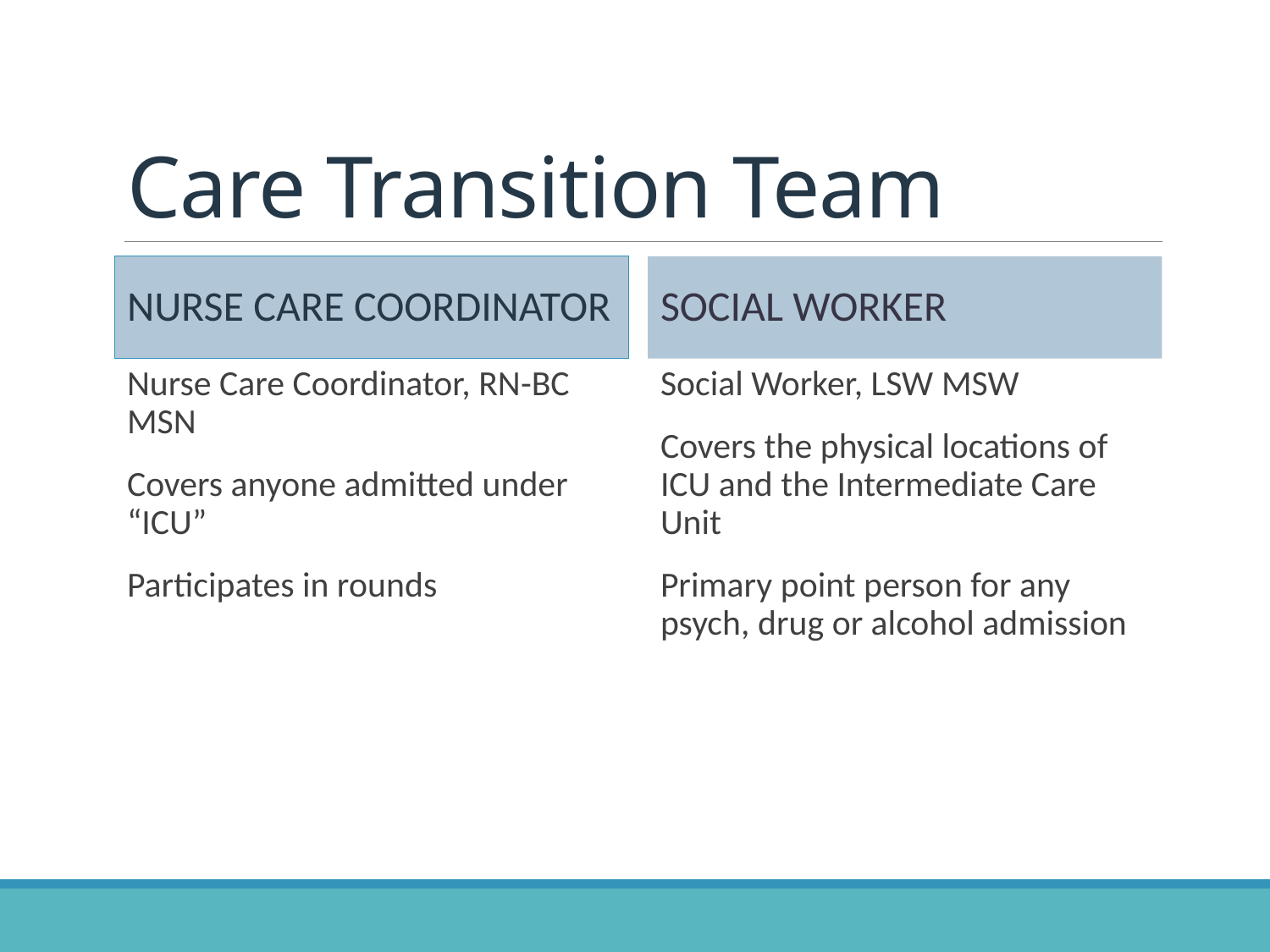

# Care Transition Team
Nurse Care Coordinator
Social Worker
Nurse Care Coordinator, RN-BC MSN
Covers anyone admitted under “ICU”
Participates in rounds
Social Worker, LSW MSW
Covers the physical locations of ICU and the Intermediate Care Unit
Primary point person for any psych, drug or alcohol admission

## Slide 5
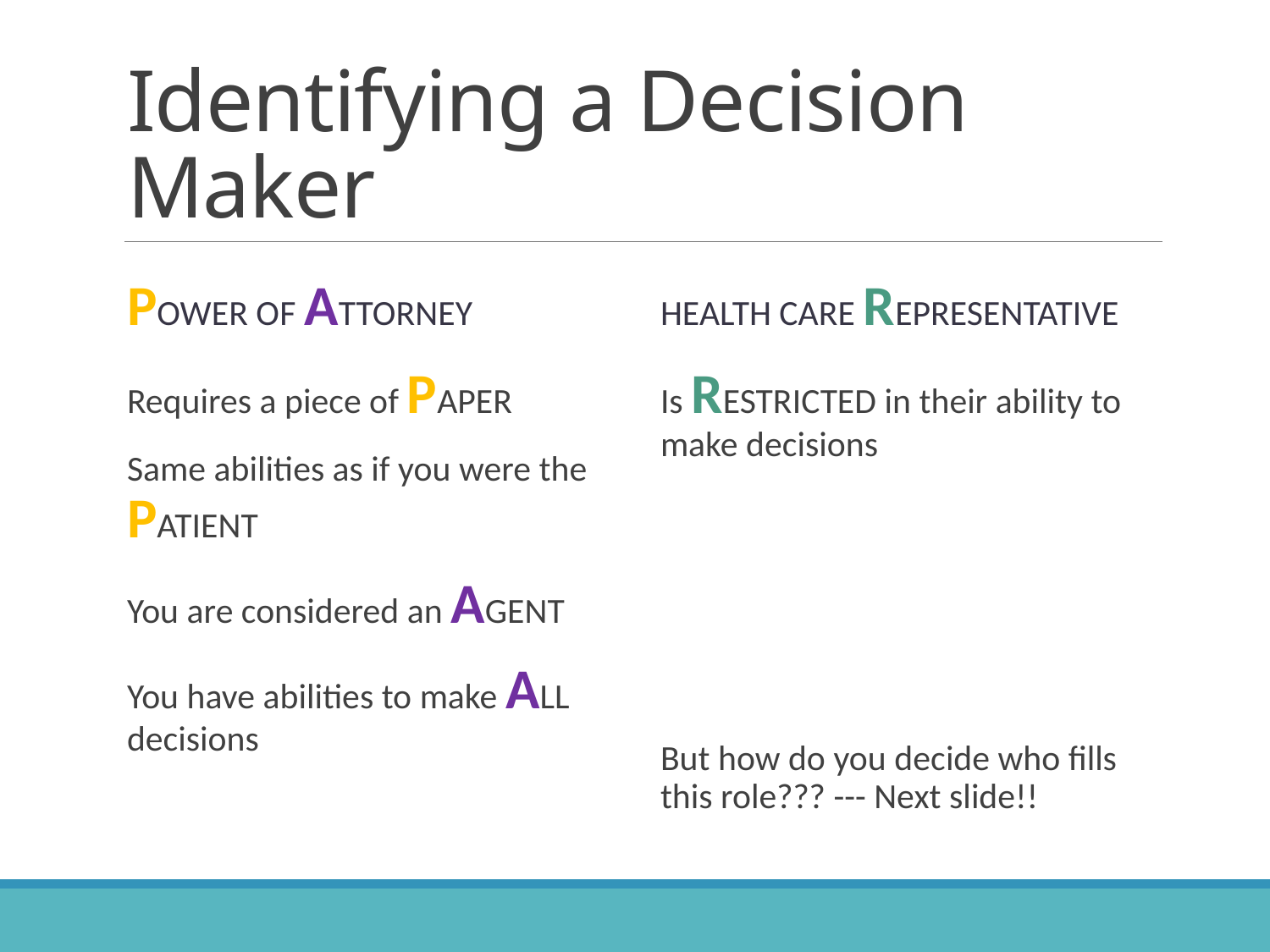

# Identifying a Decision Maker
Power of Attorney
Health Care Representative
Requires a piece of PAPER
Same abilities as if you were the PATIENT
You are considered an AGENT
You have abilities to make ALL decisions
Is RESTRICTED in their ability to make decisions
But how do you decide who fills this role??? --- Next slide!!

## Slide 6
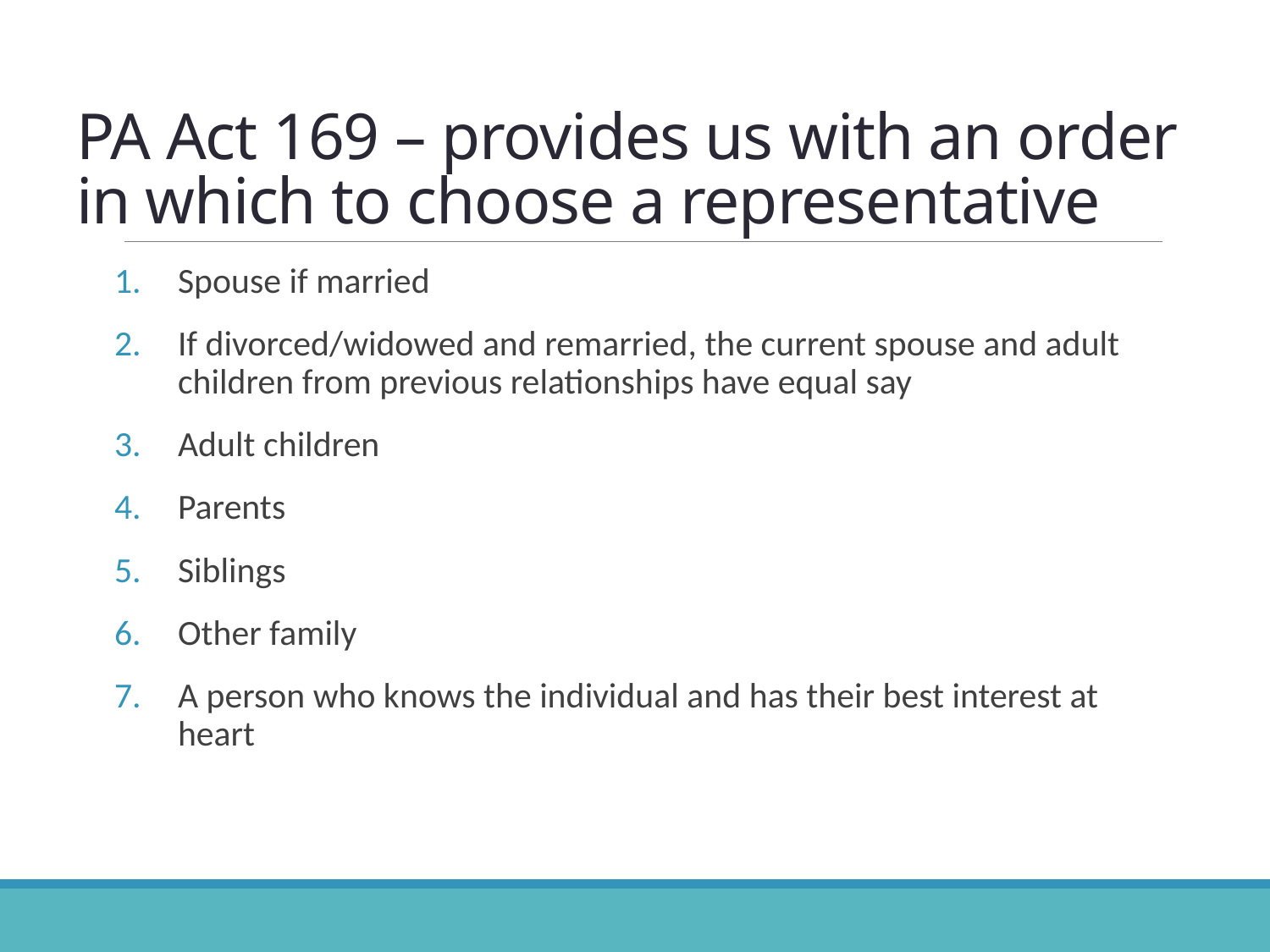

# PA Act 169 – provides us with an order in which to choose a representative
Spouse if married
If divorced/widowed and remarried, the current spouse and adult children from previous relationships have equal say
Adult children
Parents
Siblings
Other family
A person who knows the individual and has their best interest at heart

## Slide 7
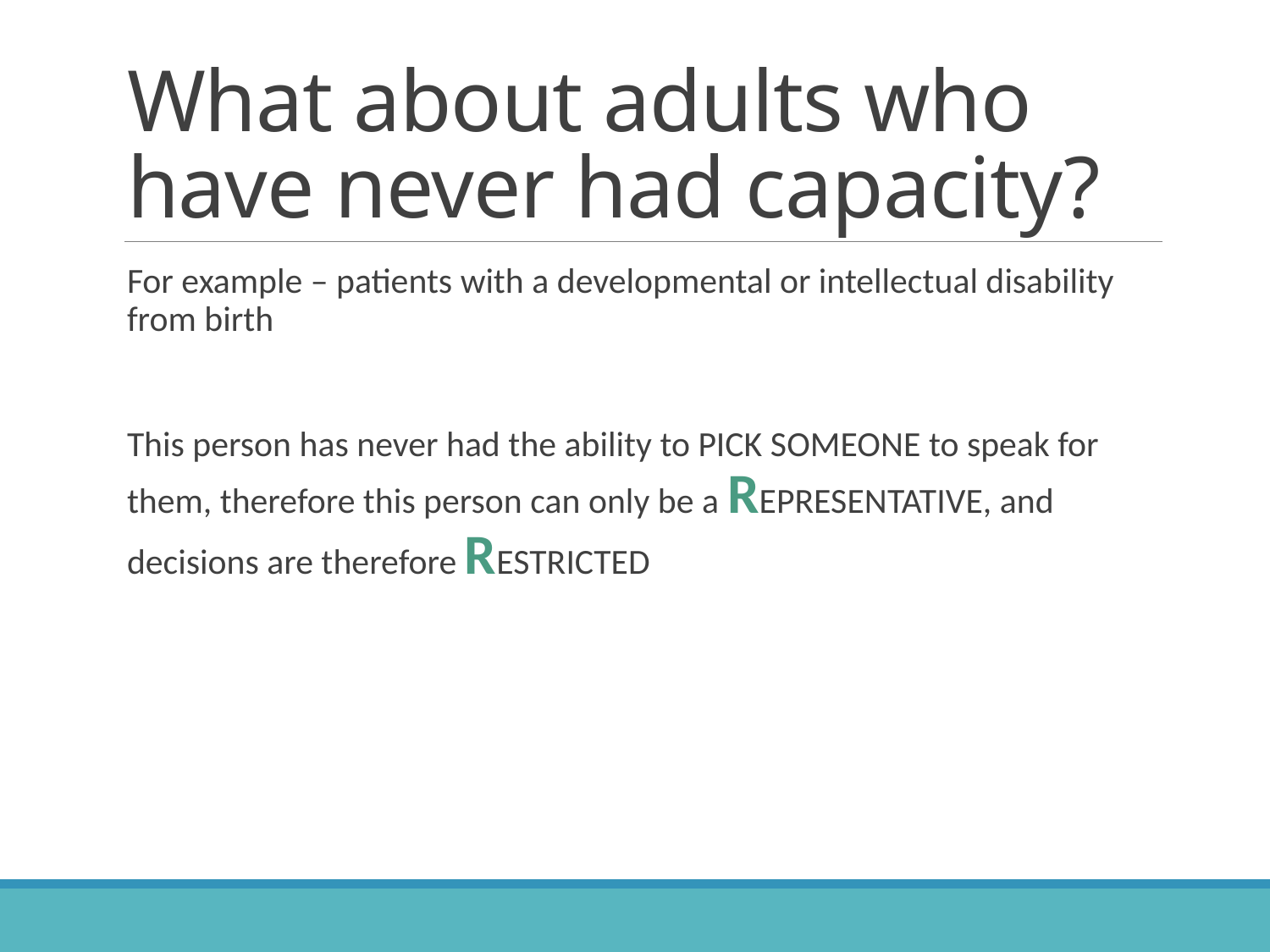

# What about adults who have never had capacity?
For example – patients with a developmental or intellectual disability from birth
This person has never had the ability to PICK SOMEONE to speak for them, therefore this person can only be a REPRESENTATIVE, and decisions are therefore RESTRICTED

## Slide 8
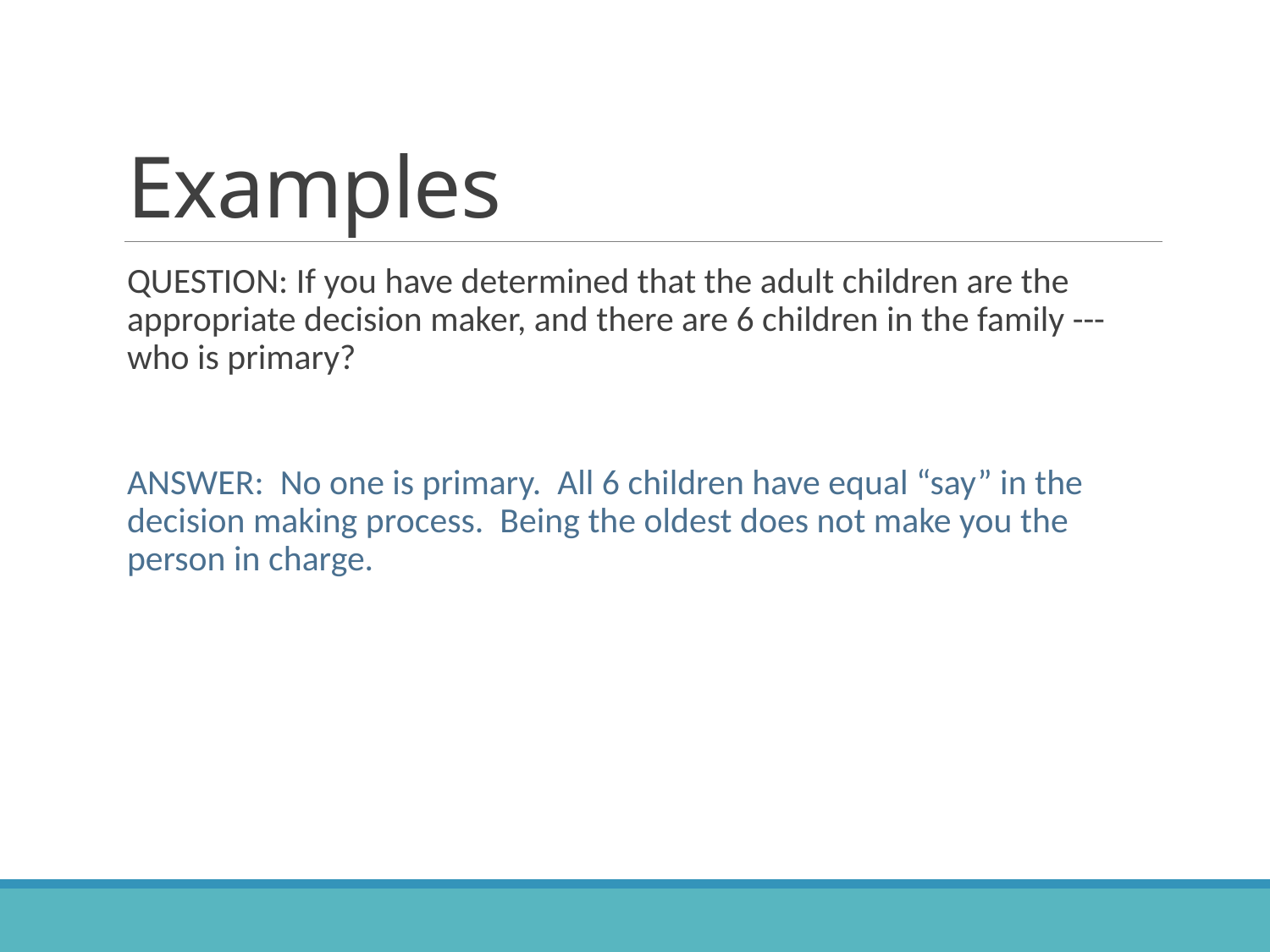

# Examples
QUESTION: If you have determined that the adult children are the appropriate decision maker, and there are 6 children in the family --- who is primary?
ANSWER: No one is primary. All 6 children have equal “say” in the decision making process. Being the oldest does not make you the person in charge.

## Slide 9
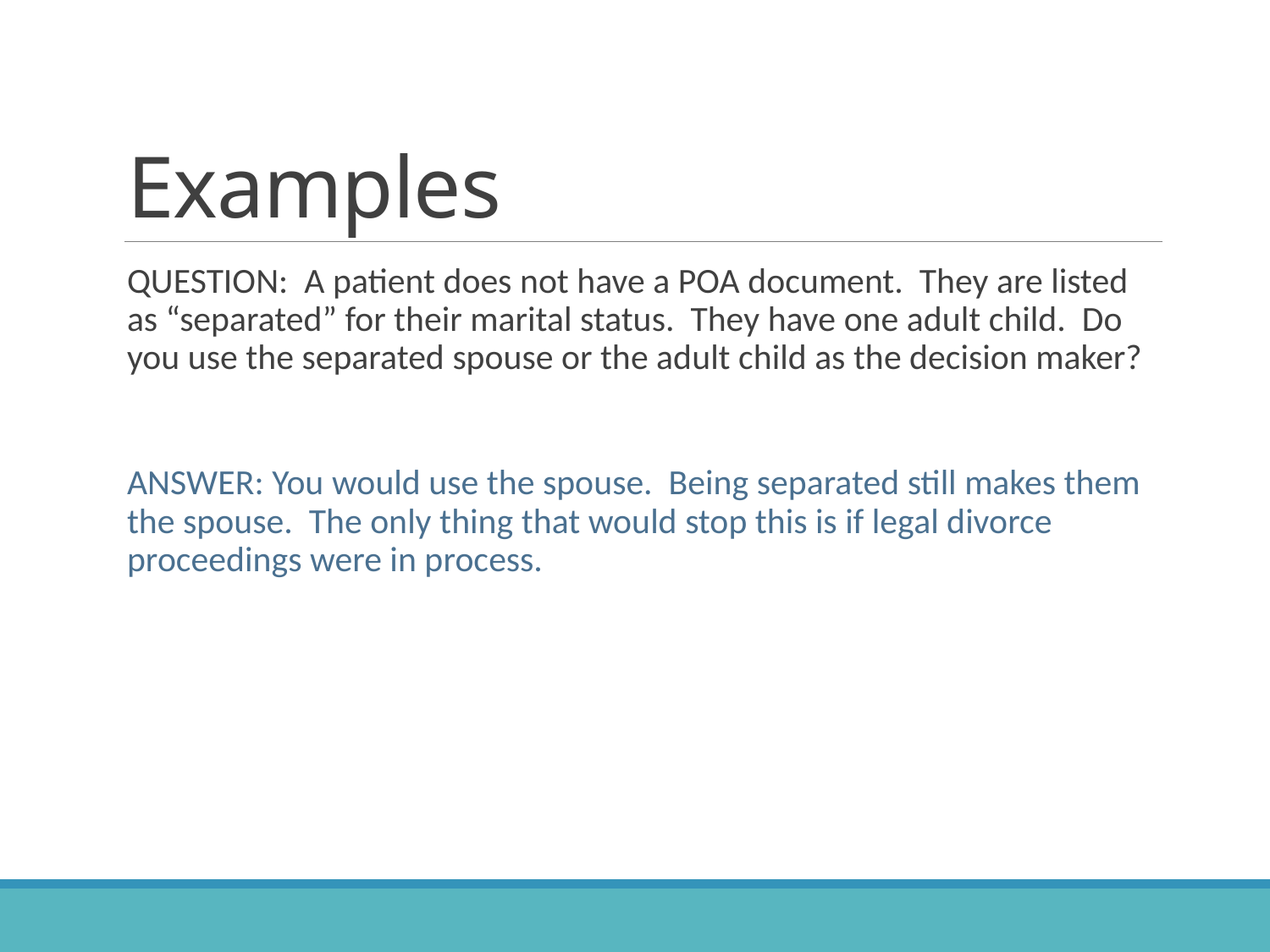

# Examples
QUESTION: A patient does not have a POA document. They are listed as “separated” for their marital status. They have one adult child. Do you use the separated spouse or the adult child as the decision maker?
ANSWER: You would use the spouse. Being separated still makes them the spouse. The only thing that would stop this is if legal divorce proceedings were in process.

## Slide 10
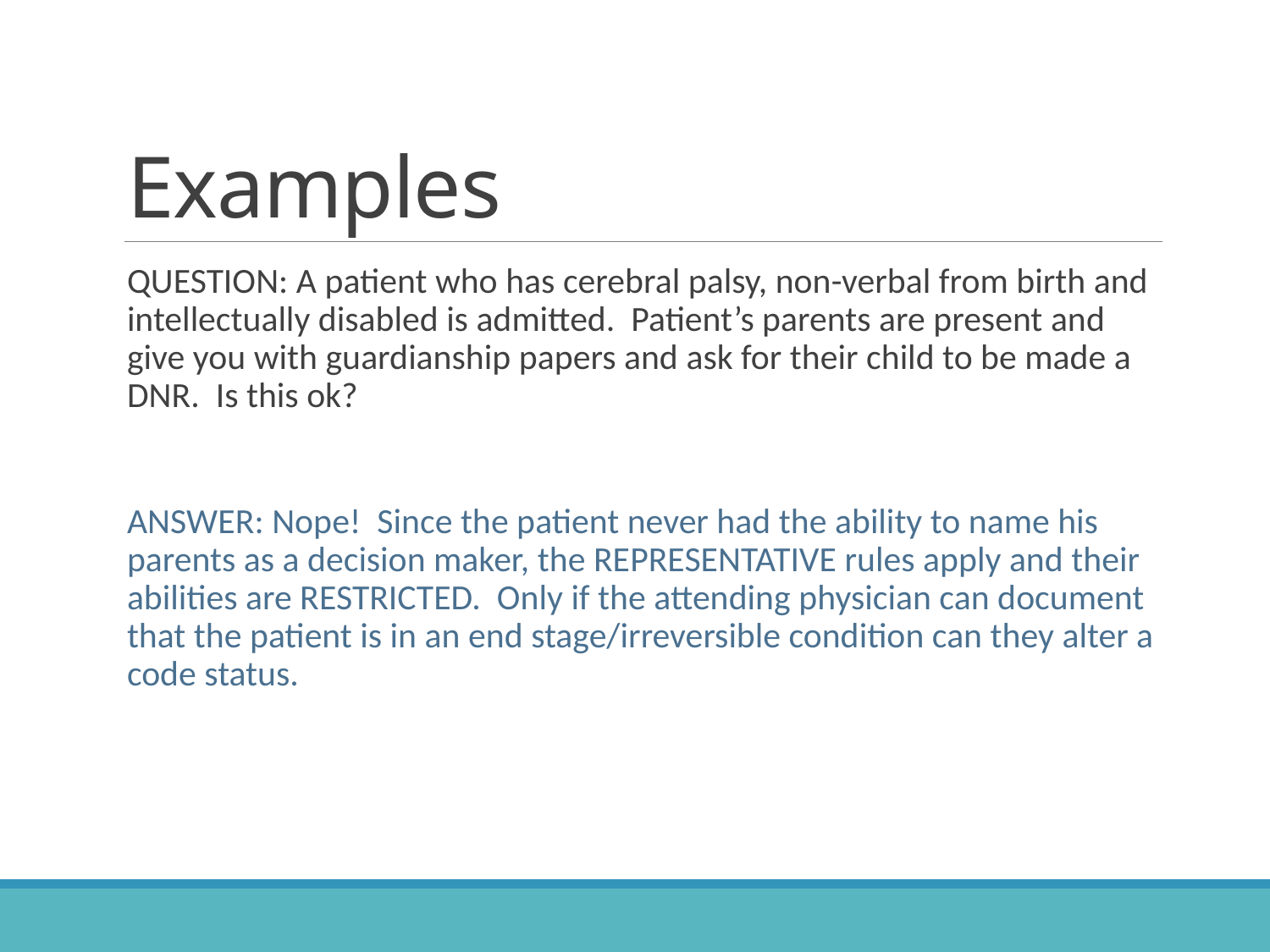

# Examples
QUESTION: A patient who has cerebral palsy, non-verbal from birth and intellectually disabled is admitted. Patient’s parents are present and give you with guardianship papers and ask for their child to be made a DNR. Is this ok?
ANSWER: Nope! Since the patient never had the ability to name his parents as a decision maker, the REPRESENTATIVE rules apply and their abilities are RESTRICTED. Only if the attending physician can document that the patient is in an end stage/irreversible condition can they alter a code status.

## Slide 11
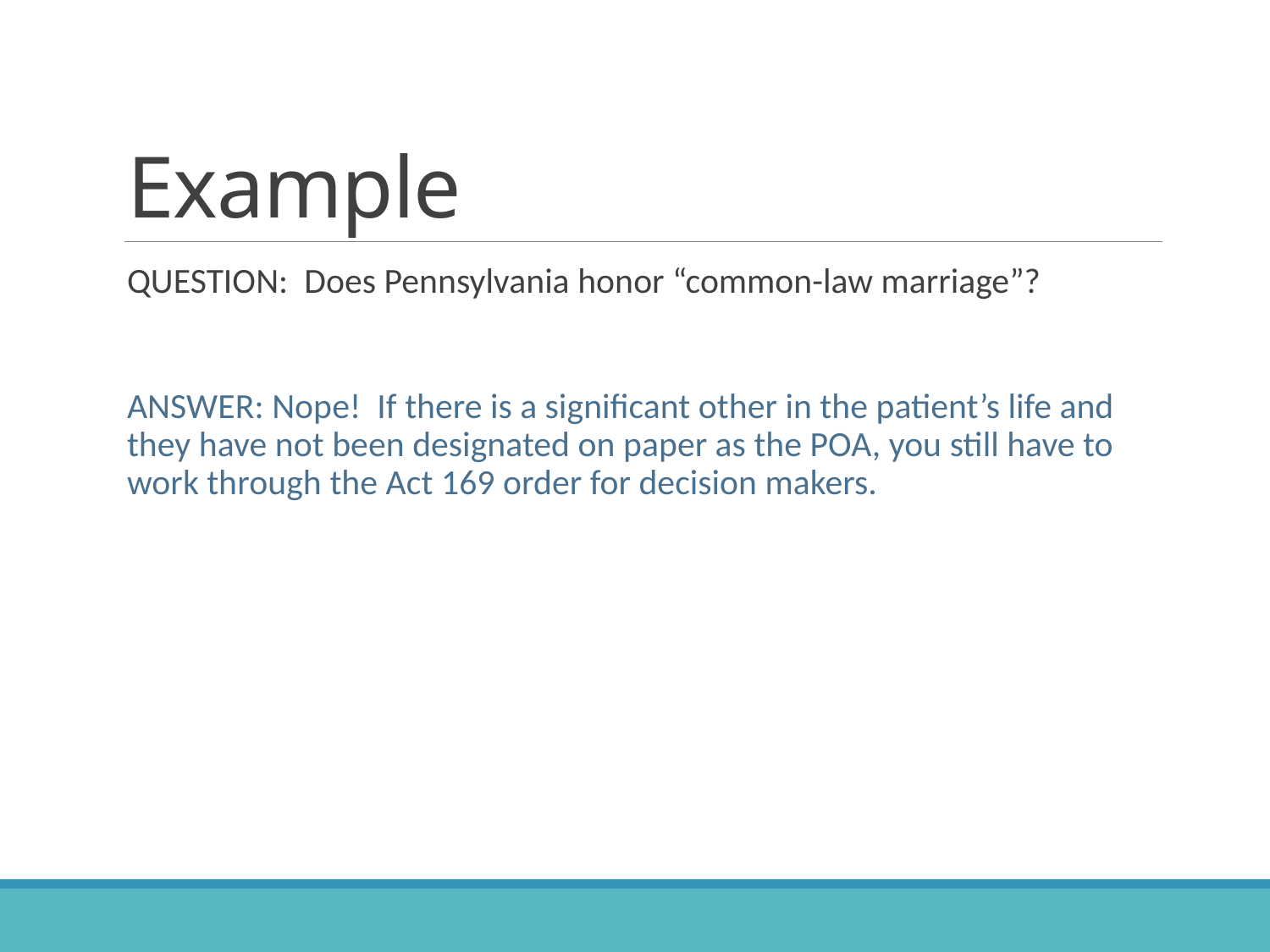

# Example
QUESTION: Does Pennsylvania honor “common-law marriage”?
ANSWER: Nope! If there is a significant other in the patient’s life and they have not been designated on paper as the POA, you still have to work through the Act 169 order for decision makers.

## Slide 12
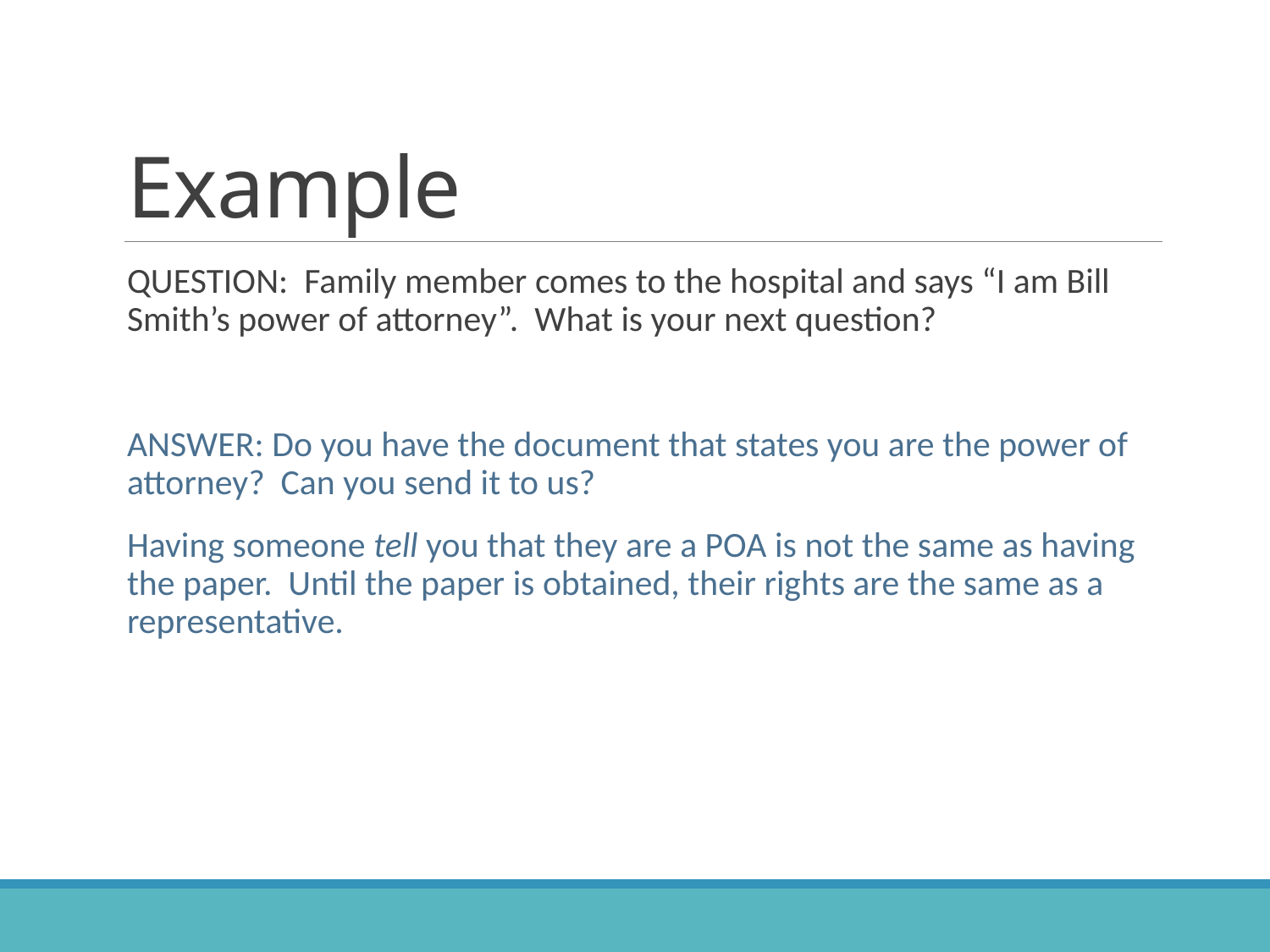

# Example
QUESTION: Family member comes to the hospital and says “I am Bill Smith’s power of attorney”. What is your next question?
ANSWER: Do you have the document that states you are the power of attorney? Can you send it to us?
Having someone tell you that they are a POA is not the same as having the paper. Until the paper is obtained, their rights are the same as a representative.

## Slide 13
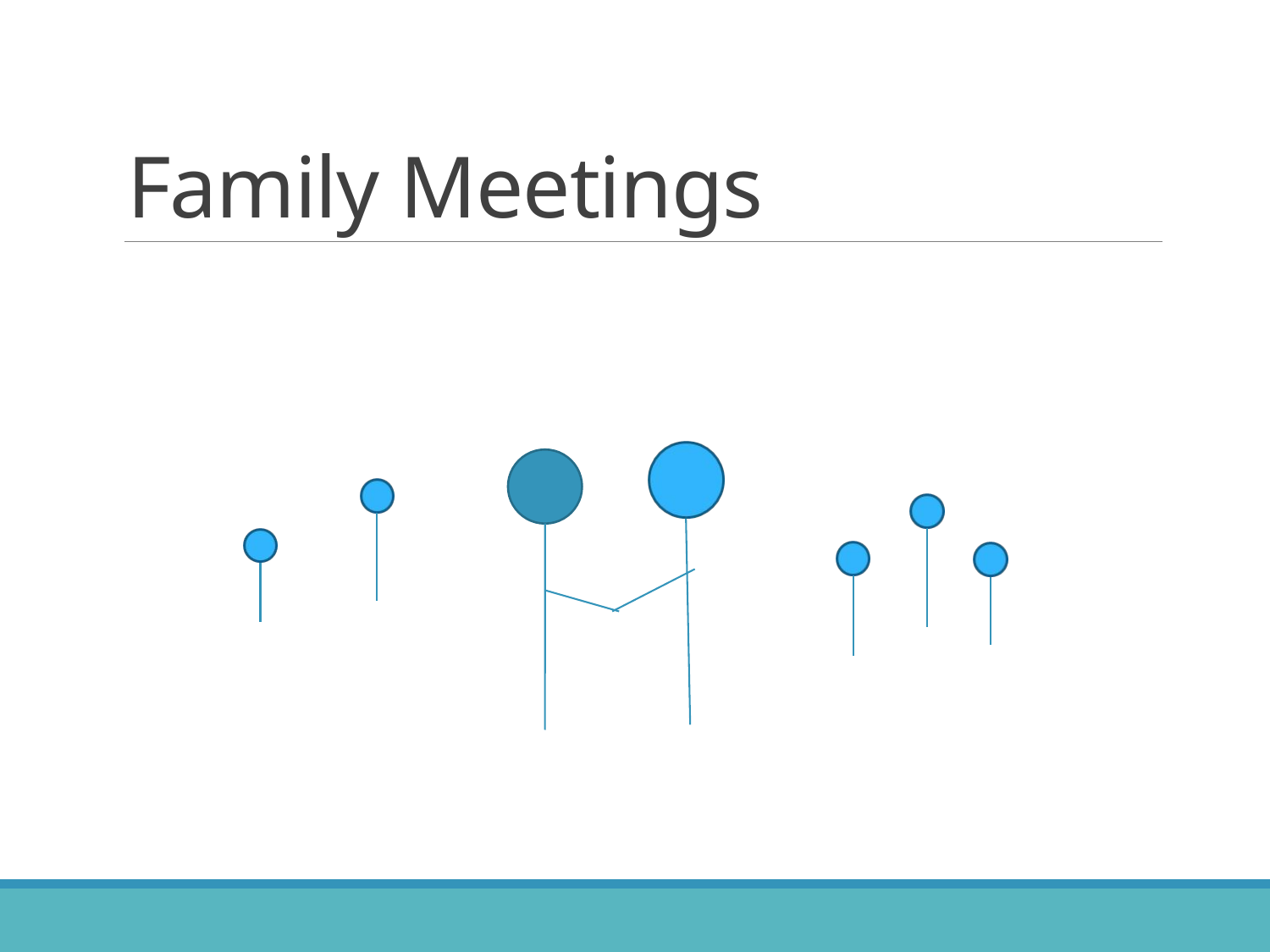

# Family Meetings

## Slide 14
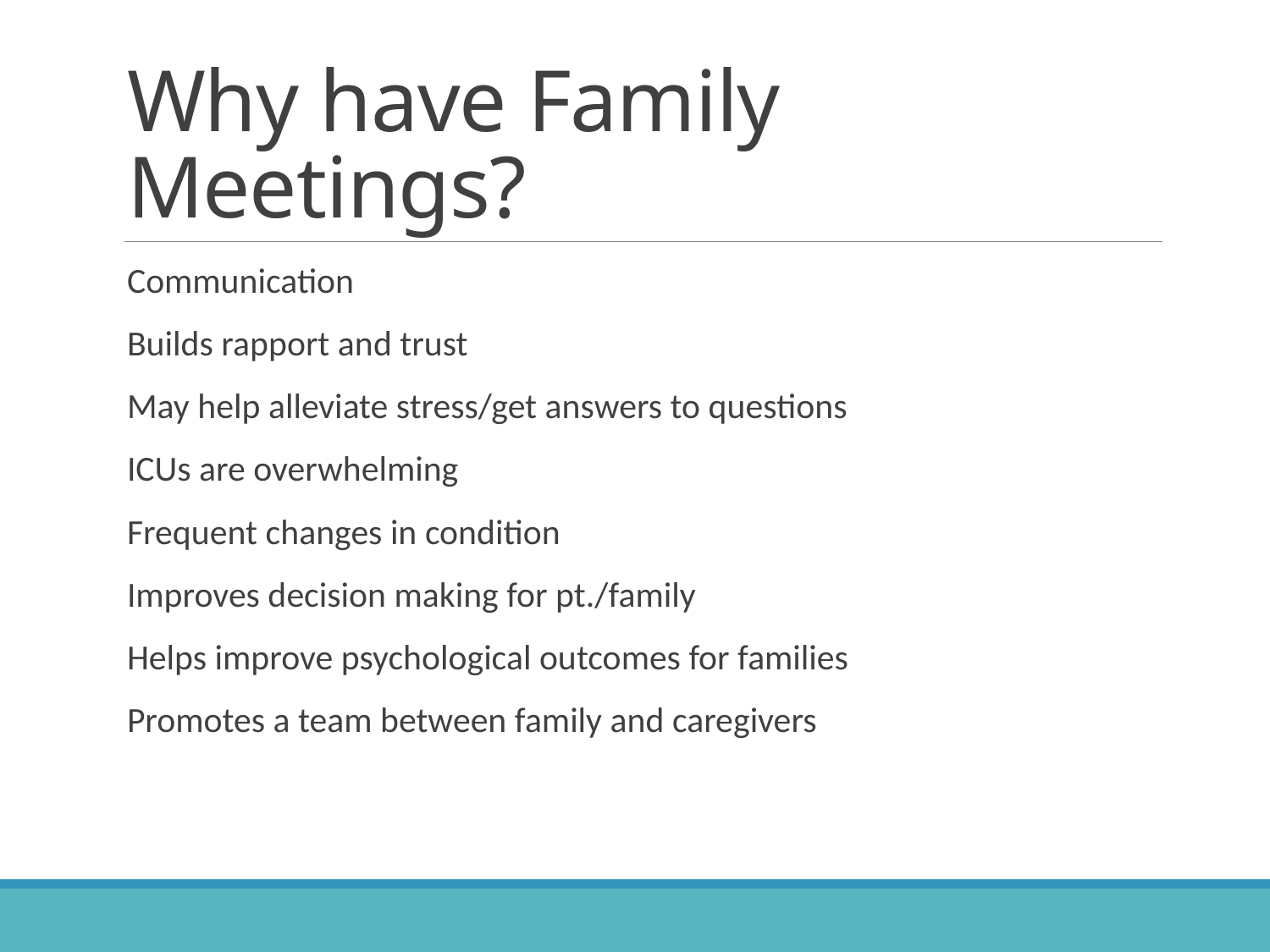

# Why have Family Meetings?
Communication
Builds rapport and trust
May help alleviate stress/get answers to questions
ICUs are overwhelming
Frequent changes in condition
Improves decision making for pt./family
Helps improve psychological outcomes for families
Promotes a team between family and caregivers

## Slide 15
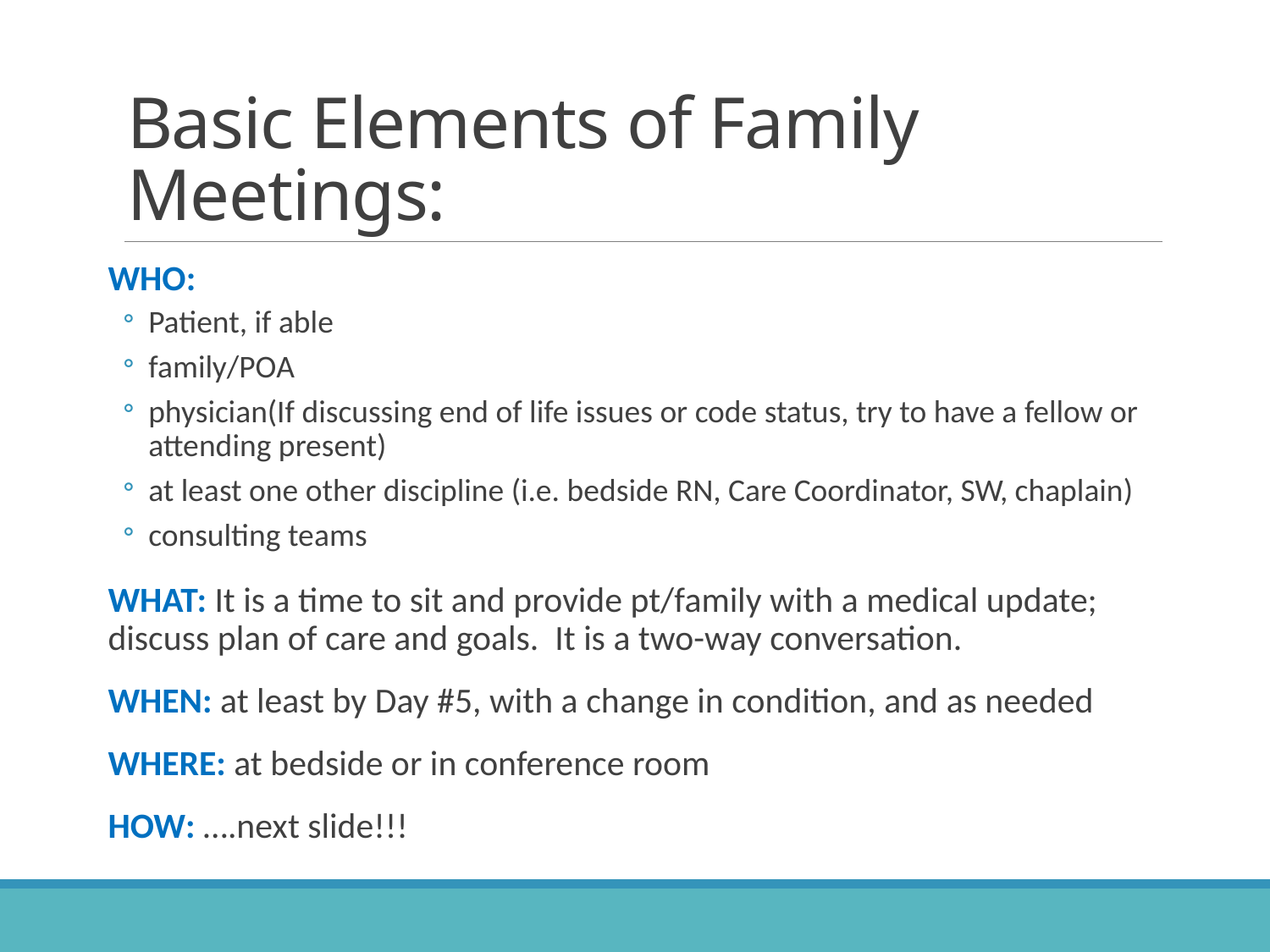

# Basic Elements of Family Meetings:
WHO:
Patient, if able
family/POA
physician(If discussing end of life issues or code status, try to have a fellow or attending present)
at least one other discipline (i.e. bedside RN, Care Coordinator, SW, chaplain)
consulting teams
WHAT: It is a time to sit and provide pt/family with a medical update; discuss plan of care and goals. It is a two-way conversation.
WHEN: at least by Day #5, with a change in condition, and as needed
WHERE: at bedside or in conference room
HOW: ….next slide!!!

## Slide 16
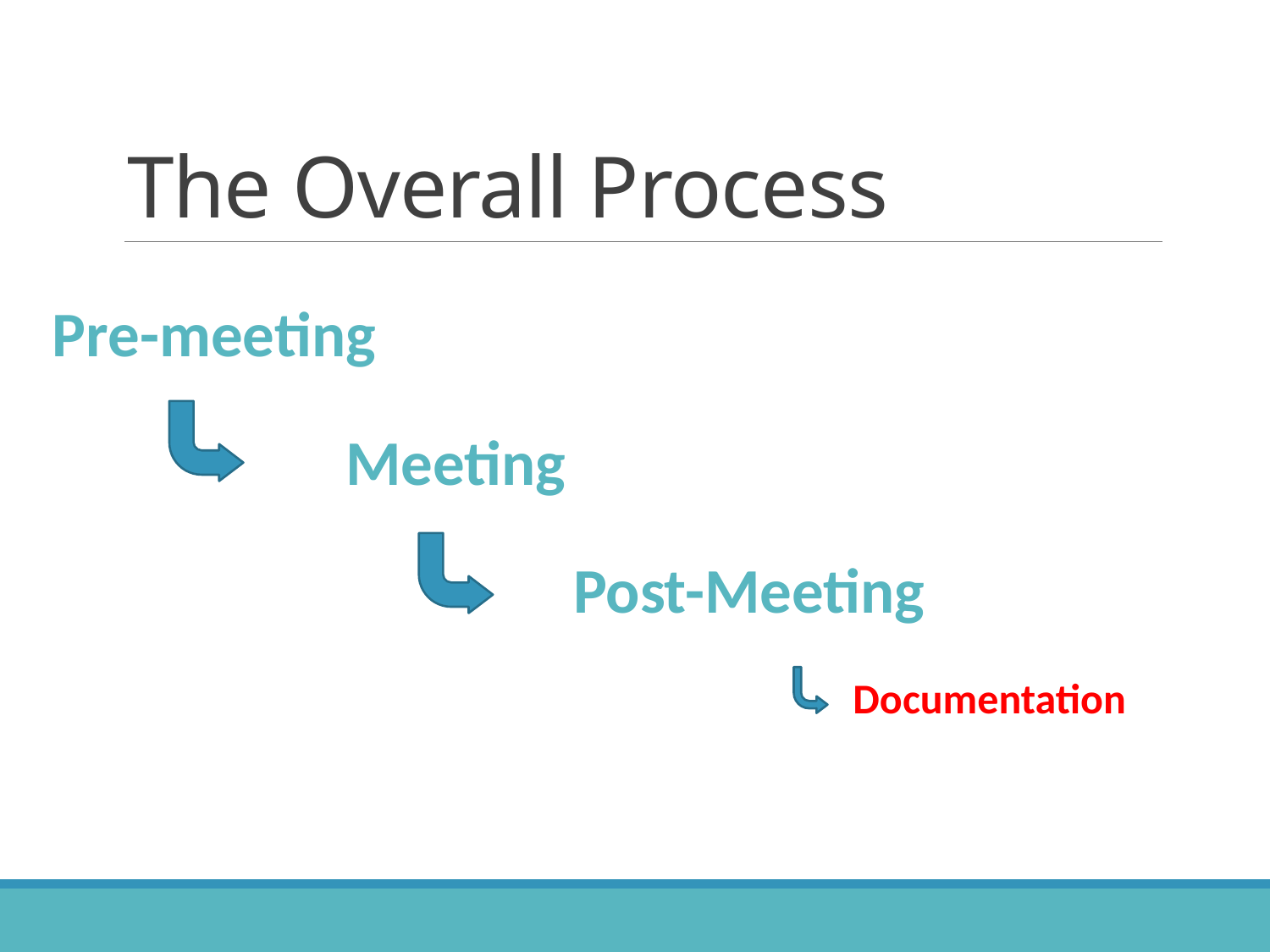

# The Overall Process
Pre-meeting
Meeting
Post-Meeting
Documentation

## Slide 17
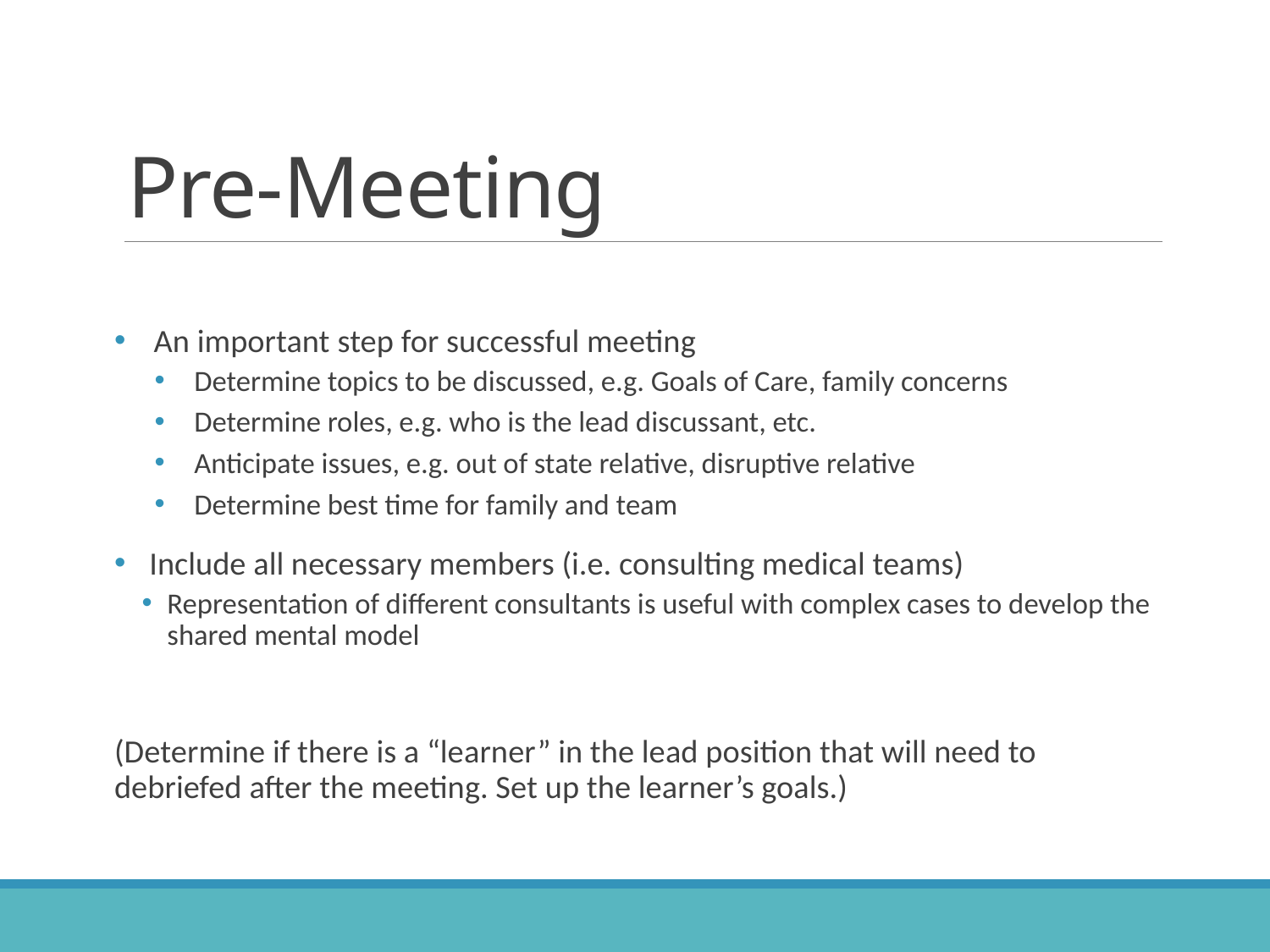

# Pre-Meeting
An important step for successful meeting
Determine topics to be discussed, e.g. Goals of Care, family concerns
Determine roles, e.g. who is the lead discussant, etc.
Anticipate issues, e.g. out of state relative, disruptive relative
Determine best time for family and team
 Include all necessary members (i.e. consulting medical teams)
Representation of different consultants is useful with complex cases to develop the shared mental model
(Determine if there is a “learner” in the lead position that will need to debriefed after the meeting. Set up the learner’s goals.)

## Slide 18
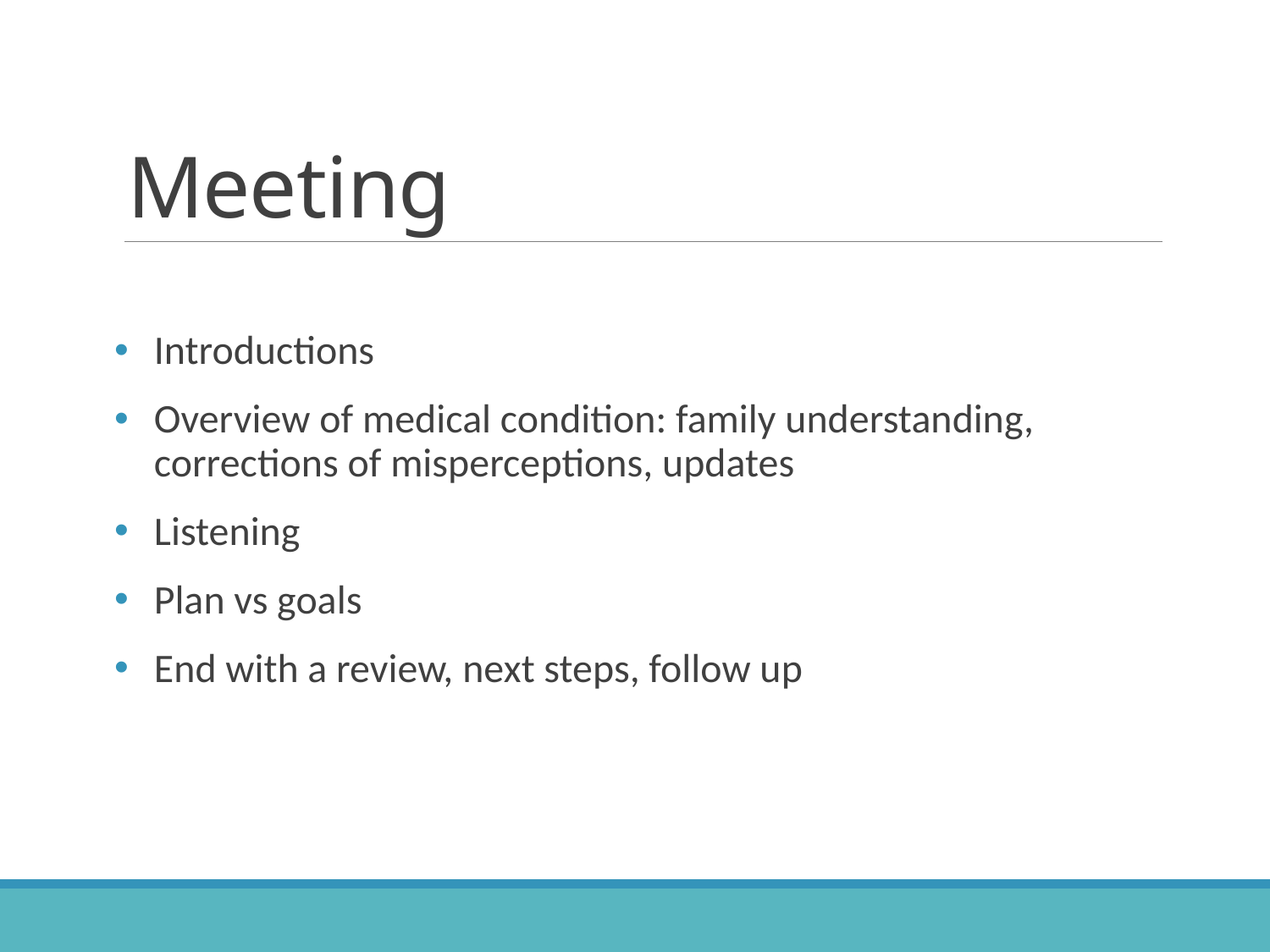

# Meeting
Introductions
Overview of medical condition: family understanding, corrections of misperceptions, updates
Listening
Plan vs goals
End with a review, next steps, follow up

## Slide 19
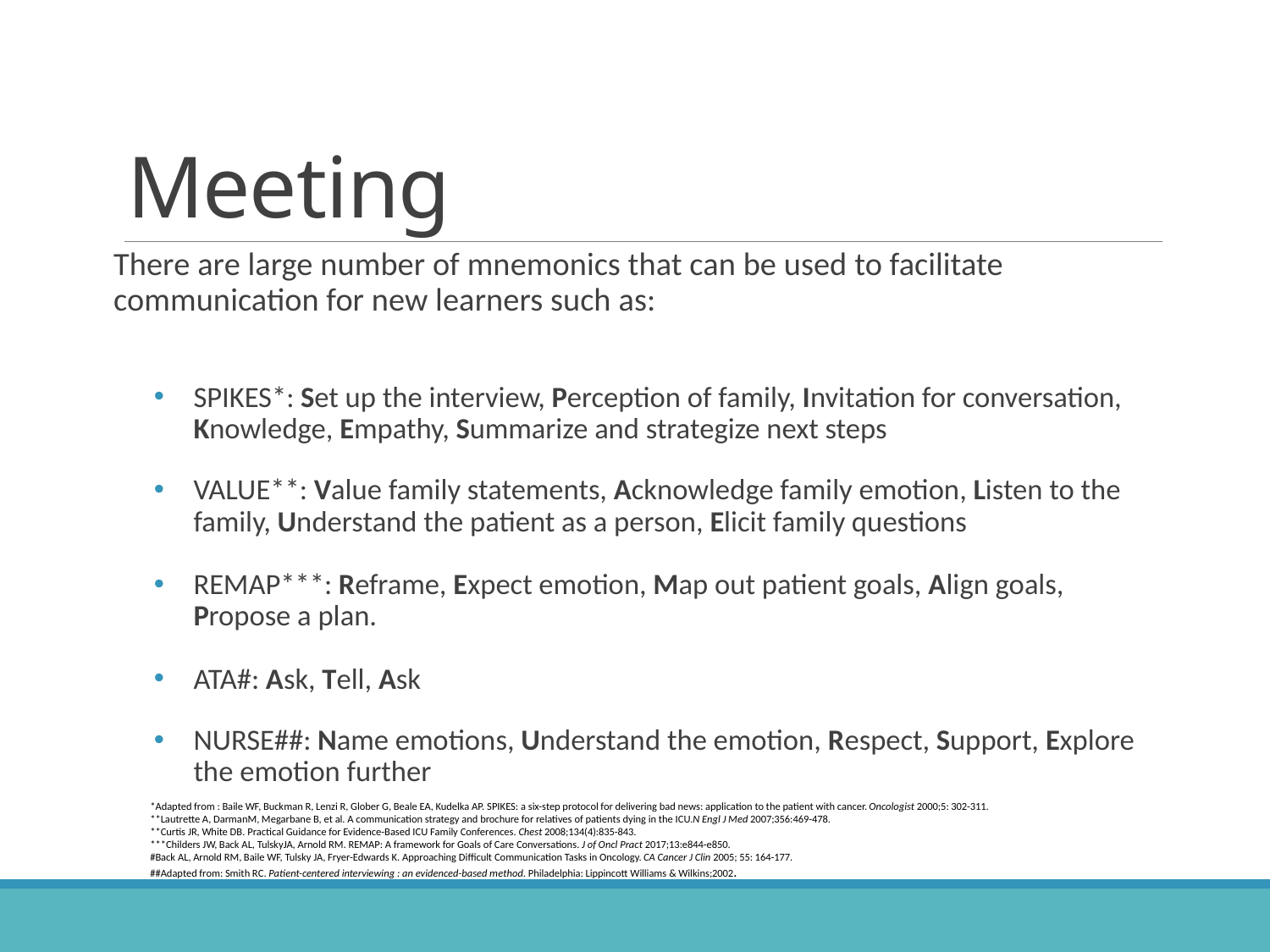

# Meeting
There are large number of mnemonics that can be used to facilitate communication for new learners such as:
SPIKES*: Set up the interview, Perception of family, Invitation for conversation, Knowledge, Empathy, Summarize and strategize next steps
VALUE**: Value family statements, Acknowledge family emotion, Listen to the family, Understand the patient as a person, Elicit family questions
REMAP***: Reframe, Expect emotion, Map out patient goals, Align goals, Propose a plan.
ATA#: Ask, Tell, Ask
NURSE##: Name emotions, Understand the emotion, Respect, Support, Explore the emotion further
*Adapted from : Baile WF, Buckman R, Lenzi R, Glober G, Beale EA, Kudelka AP. SPIKES: a six-step protocol for delivering bad news: application to the patient with cancer. Oncologist 2000;5: 302-311.
**Lautrette A, DarmanM, Megarbane B, et al. A communication strategy and brochure for relatives of patients dying in the ICU.N Engl J Med 2007;356:469-478.
**Curtis JR, White DB. Practical Guidance for Evidence-Based ICU Family Conferences. Chest 2008;134(4):835-843.
***Childers JW, Back AL, TulskyJA, Arnold RM. REMAP: A framework for Goals of Care Conversations. J of Oncl Pract 2017;13:e844-e850.
#Back AL, Arnold RM, Baile WF, Tulsky JA, Fryer-Edwards K. Approaching Difficult Communication Tasks in Oncology. CA Cancer J Clin 2005; 55: 164-177.
##Adapted from: Smith RC. Patient-centered interviewing : an evidenced-based method. Philadelphia: Lippincott Williams & Wilkins;2002.

## Slide 20
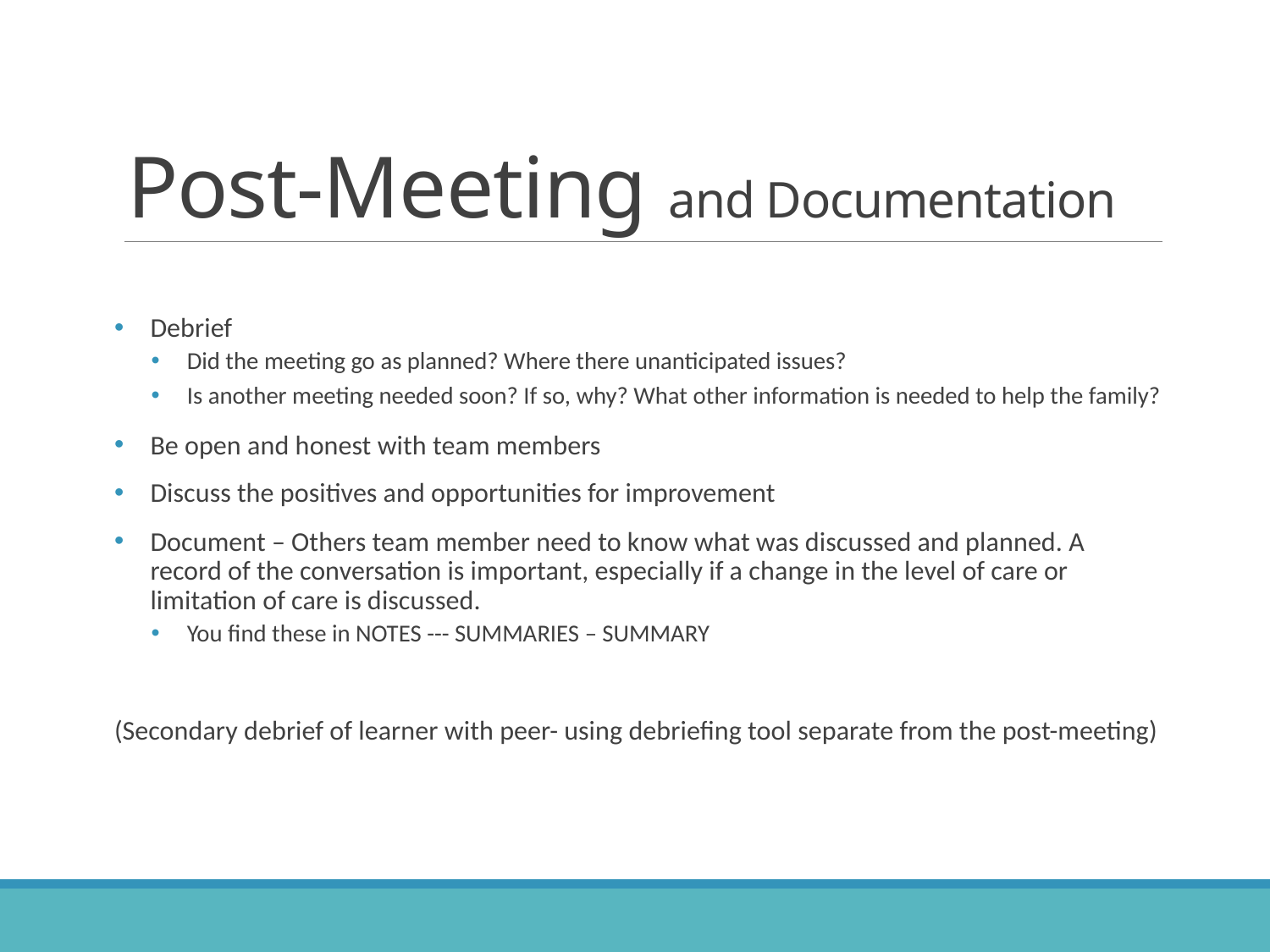

# Post-Meeting and Documentation
Debrief
Did the meeting go as planned? Where there unanticipated issues?
Is another meeting needed soon? If so, why? What other information is needed to help the family?
Be open and honest with team members
Discuss the positives and opportunities for improvement
Document – Others team member need to know what was discussed and planned. A record of the conversation is important, especially if a change in the level of care or limitation of care is discussed.
You find these in NOTES --- SUMMARIES – SUMMARY
(Secondary debrief of learner with peer- using debriefing tool separate from the post-meeting)

## Slide 21
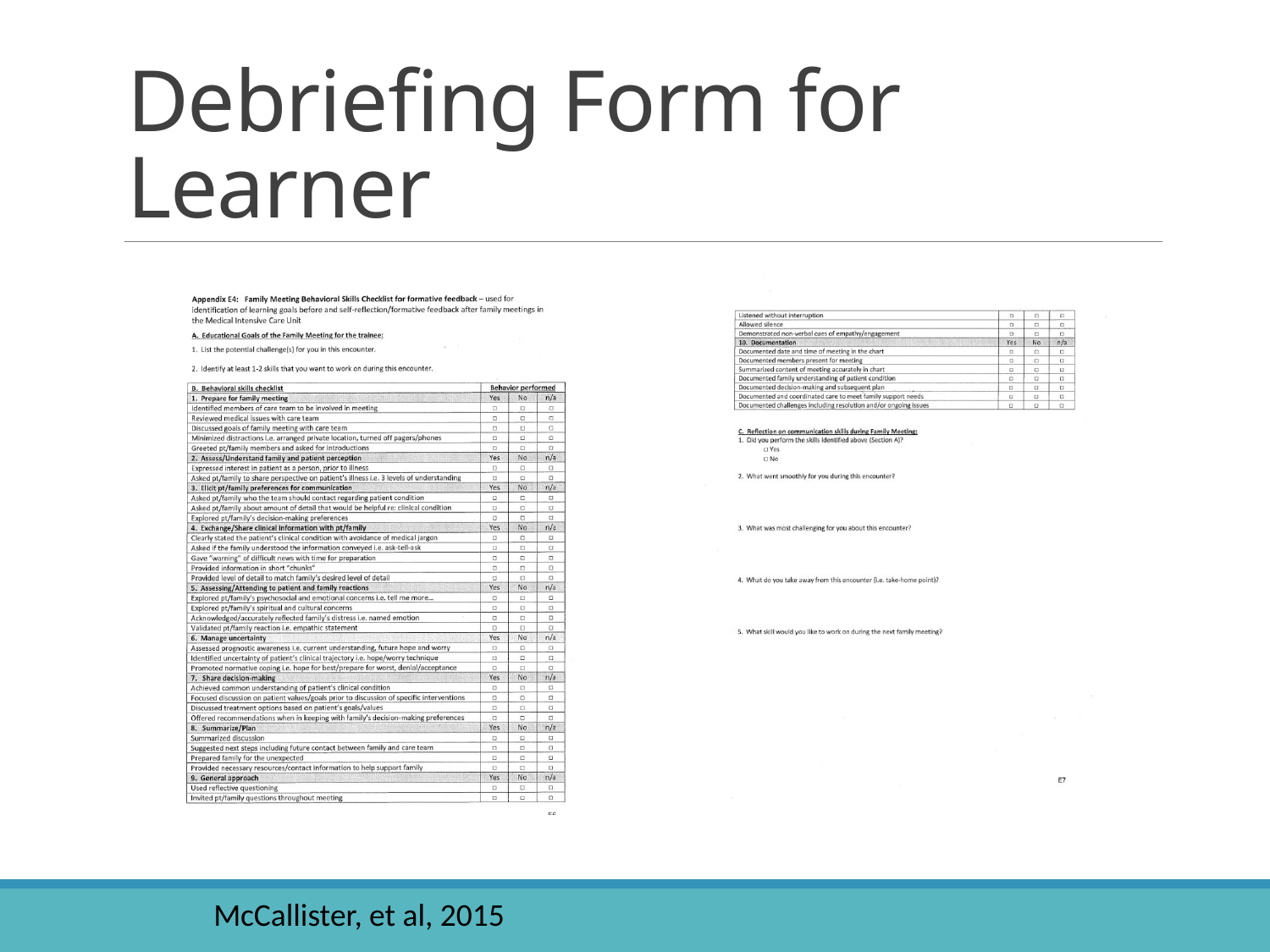

# Debriefing Form for Learner
McCallister, et al, 2015

## Slide 22
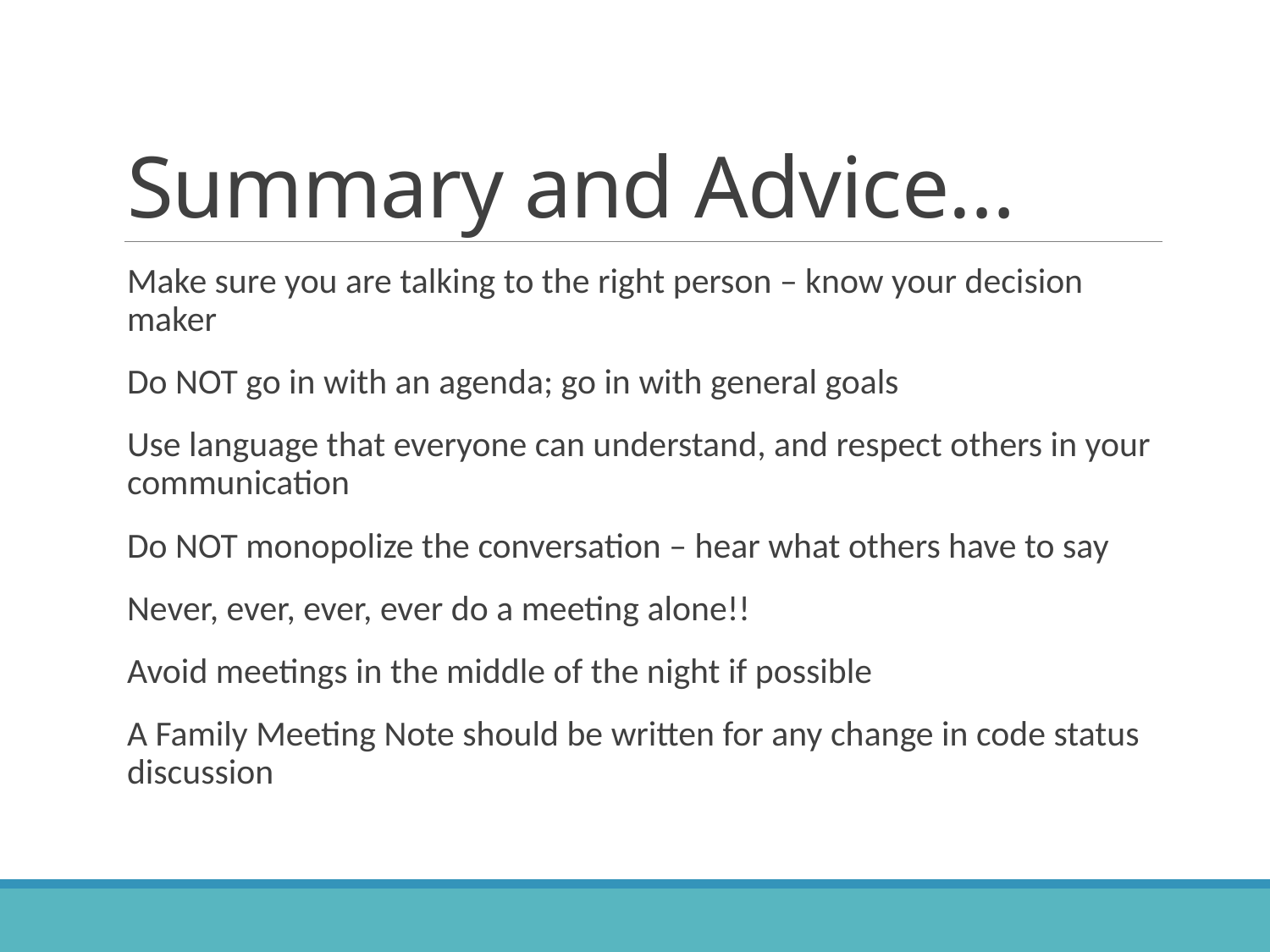

# Summary and Advice…
Make sure you are talking to the right person – know your decision maker
Do NOT go in with an agenda; go in with general goals
Use language that everyone can understand, and respect others in your communication
Do NOT monopolize the conversation – hear what others have to say
Never, ever, ever, ever do a meeting alone!!
Avoid meetings in the middle of the night if possible
A Family Meeting Note should be written for any change in code status discussion
