# Supplementary material for: Family Meeting Training Curriculum: A Multimedia Approach With Real-Time Experiential Learning for Residents
Source: MedEdPORTAL. 2020 Mar 6;16:10883. doi: 10.15766/mep_2374-8265.10883 (PMC7062545; doi:10.15766/mep_2374-8265.10883)
Supplement: Supplementary file 1 — A. Communication Basics.pptx B. Family Meeting E-Learning Project folder C. ICU Resident Orientation.pptx D. Family Meeting Resources Booklet.docx E. FMBS Tool.docx F. Global Self-Efficacy Survey.docx [file mep-16-10883-s001.zip › D. Family Meeting Resources Booklet.docx]

Family Meeting

Reference Guide

CONTACTS

Palliative Medicine

Physician Unit Champion

Social Worker

Care Coordinator

Nursing Unit Champion

Pastoral Services

**FAMILY MEETING/CONFERENCE: BLUEPRINT for SUCCESS**

**PRE-MEETING**

1. Gather the Facts – Be Informed

- Review chart.

-Know all medical issues: treatment course, prognosis, treatment options.

- Coordinate medical opinions among consultant physicians.

-Decide what tests/treatments are medically appropriate.

- Review Advance Care Planning documents.
- Review/obtain family psychosocial information.

2. Establish Meeting Goals

- Check your own emotions.

3. Determine Meeting Venue/Participants – Be Prepared

- Ensure venue is private, comfortable and accommodates sitting in a circle.
- Identify participants – at least two disciplines should be represented.
- Identify background information and/or staff concerns.
- Identify main facilitator.
- Set time and place.
- Notify all participants.

**MEETING ESSENTIALS**

1. Perform introductions.

2. State ground rules.

3. Explain reason/goals for meeting.

4. Elicit family understanding.

5. Explore the “how” of the patient situation.

6. Explore family view of patient wishes under the circumstances.

7. Summarize case (avoid jargon).

8. Solicit family summary (give family time to speak) and clarify, if necessary.

9. LISTEN (note time spent).

10. Frame recommendation(s) and goals.

11. Explore concerns.

12. Recognize/address conflict.

13. Summarize meeting.

14. Provide plan for follow-up.

15. Review “next steps.”

**POST-MEETING DEBRIEF**

1. Allow team members to speak.

2. Assess: What went well? What can we do differently?

3. Review uncomfortable moments and/or surprises.

4. Review how the family is coping.

5. Ask about perceptions of the teamwork.

6. Discuss residual concerns.

7. Document meeting discussion on Family/Patient Meeting Summary in electronic health record (EHR).

**HELPFUL TIPS**

1. Establishing Introductions/Goals/Relationship

Invite everyone to state name and relationship to patient.

- Identify who might be a legal decision maker (e.g., POA, guardian).
- State your meeting goals, ask family members to state their goals and identify joint goals.
- Build relationship: ask nonmedical questions about the patient, such as “*What kind of things does your father enjoy?”*

2. Seeking Family Understanding of Medical Condition and Preferences for Sharing/Decision-Making

*•* Use language such as “*Tell me your understanding of [family member’s name] current medical condition.”*

• Encourage all present to respond.

• Explore what it is like for the patient now.

• For patients with chronic illness, ask for description of changes

in function over past weeks or months (activity, eating, sleep,

mood), such as:

“*How have things been going during the past three months?”*

3. Providing Medical Review/Summary

• Summarize “the big picture” in a few sentences. Avoid organ-by-organ

medical review.

• Don’t use jargon. Answer questions.

• Explore family’s notions about what the patient would want, such as:

“*What would your father want under these circumstances?*”

4. Embracing Silence/Reactions

• Allow for silence, especially if the family seems conflicted.

-This allows for family expression.

-Silence signals you are listening.

5. Affirming Emotions

• Respond to emotional reactions.

• Prepare for common reactions: acceptance, conflict, denial, grief,

despair, anger, guilt.

• Respond empathetically to conflict/denial.

*“I can’t imagine how hard this is for you.”*

6. Framing Recommendations: Present Broad Care Options/Set Goals

• Provide prognostic data using a range.

• Respond to emotions.

• Present goal-oriented options (e.g., prolong life, improve

function, return home, dignified death).

• Stress priority of comfort, no matter the goal.

• Make a recommendation based on knowledge/experience.

7. Translating Goals into Care Plan

• Review current and planned interventions; make

recommendations to continue or stop based on goals.

• Summarize all decisions made.

• Translate goals and decisions into appropriate order for level of

support (i.e., POLST, referral to a facility, home care, hospice).

8. Documenting and Discussing

• Write a note describing who was present, what decisions were

made, follow-up plans, etc.

• Discuss situation with team members (consultants, nurse, etc.);

check your emotions; participate in a team debriefing.

9. Demonstrating Empathetic Behaviors and Language

• Normalize/validate: *“It is normal to be upset at such a difficult*

*moment.”*

• Name or acknowledge the emotion: “*You seem sad.” “I can see*

*you are upset.”*

• Gesture/touch: Offer tissues, a drink, a gentle touch (if

culturally appropriate).

• Encourage expression: “*Tell me more about how you are feeling.”*

• Paraphrase/repeat back: “*If I understand you correctly…”*

• Empathize: *“I wish things were different.”*

• Praise/affirm: *“You are being a strong voice for him.”*

• Silence is also an empathetic behavior that reflects listening, acknowledging and sharing suffering.

10. Managing Conflict

• Listen and make empathetic statements.

• Determine source of conflict: guilt, grief, culture, family

dysfunction, trust in medical team, etc.

• Clarify misconceptions; explore values behind decisions.

• Set time-limited goals with specific benchmarks (e.g., improved

cognition, oxygenation and mobility).

**COMMUNICATION TIPS: PHRASES FOR SPECIFIC CIRCUMSTANCES**

**Advance Care Planning**

“I’d like to talk with you about possible health care decisions in the

future. This is something I do with all my patients so I can be sure

I know and can follow your wishes. Have you ever completed an

Advance Directive?”

“What do you understand about your health situation?”

“If you were unable to make your own medical decisions, who

would you like to make decisions for you? Have you spoken to this

person about your wishes?”

“When you think about dying, have you thought about what the end

would be like or how you would like it to be?”

“Have you discussed your wishes with your family?”

**Determining Decision-Making Capacity**

“Will you describe your current condition? What have the doctors

told you?”

“Tell me the options we have just discussed for treating X.”

“Explain to me why you feel that way.”

**Talking with Surrogate Decision Makers**

“These decisions are very hard; if (patient’s name) were sitting with us

now, what do you think he or she would say?”

“Can you tell me why you feel that way?”

“How will the decision affect you and other family members?”

“I believe that (patient’s name) is dying.”

**Cross-Cultural: Understanding Others’ Views of Illness**

“I know different people have very different ways of understanding

illness. Please help me understand how you see things.”

“What do you call the problem? Tell me what you think the illness

does. What do you think the natural course of the illness is? What do

you fear?”

“Who do you turn to for help? Who should be involved in decision-making?”

“How do you think your condition should be treated? How do you want

us to help you?”

“Some people like to know everything about their disease and be

involved in all decision-making. Others do not want all the news and

would rather the doctor talk to a loved one). Which kind of person are

you? How involved do you want to be in these decisions?”

**Quality of Life: Phrases That Will Help You Understand the Illness’s Impact**

“How has your disease interfered with your daily activities? With your

family and friends?”

“Have you been feeling worried or sad about your illness?”

“What symptoms bother you the most? What concerns you the most?”

“How have your religious beliefs been affected by your illness?”

“Many patients wonder about the meaning of all this—do you?”

“Tell me how you spend your day? How much time do you spend

lying down or resting—is it more or less than 50 percent of the time? Has

this changed recently?”

**Discussing Prognosis**

“Has anyone talked to you about what to expect?”

“Do you have any sense of how much time is left? Is this something

you would like to talk about?”

“Although I can’t give you an exact time, in general, patients with your

condition live __ weeks/months to __ weeks/months.”

“Based on what you have told me, and what I see, I believe you are

dying.”

**Breaking Bad News**

Ask for permission first. “I’m afraid I have bad news. Is it okay if we

talk about it now?”

“What do you understand about your condition?”

“I’m afraid I have some bad news. I wish things were different, but the

test results are not good. The (test name) showed (result).”

**Discussing Artificial Feeding/Hydration**

“What do you know about artificial ways to provide food?”

“All dying patients lose their interest in eating in the days to weeks

leading up to death. This is the body’s signal that death is coming.”

“I am recommending that the (tube feedings, hydration, etc.) be

discontinued (or not started) as these will not improve his/her life.”

“Your (relation) is dying from (disease). He or she is not dying from

dehydration or starvation.”

**Discussing Palliative Care or Hospice Referral**

“To meet the goals we’ve discussed (summarize goals), I’ve asked the

Palliative Care Team to visit with you. They are experts in treating the

symptoms you are experiencing. They can help your family deal with

the changes brought on by your illness.”

“You’ve told me you want to be as independent and comfortable as

possible. Hospice care is the best way I know to help you achieve

those goals. It’s a program that helps the patient and family

achieve the goals you’ve just described. The hospice team helps meet

the patient’s and family’s physical, psychological, social and spiritual

needs.”

**How to Discuss DNR with Limited Support**

**NOTE:** Only discuss CPR/DNR following a goal-setting discussion

1. When CPR is not consistent with goals.**

“You have told me your goals are _____________. With this

in mind, I do not recommend the use of artificial or heroic means

to keep you alive. If you agree with this, I will write an order in

the chart that when you die, no attempt to resuscitate you will be

made. Is this acceptable (okay)?”

- - This will allow a natural death and continue to focus on measures

to ensure comfort.

2. When CPR is medically indicated, if consistent with patient

goals/wishes.

“We have discussed your current illness. Have you given any

thought to how you would like to be cared for at the time of

death? Sometimes when people die or are near death, especially

from a sudden illness, life support measures are used to try to

‘bring them back.’ Alternatively, we could focus solely on keeping

you comfortable. How do you feel about this?”

- All discussions regarding withdrawal of support must include two

disciplines and, preferably, a fellow or attending. If fellow or attending are not readily available or not on-site, they must be

informed prior to placing an order for withdrawal.

- Avoid discussions of withdrawal of support overnight. If needed,

**emergently** inform the on-call fellow or attending.

3. Sustained requests for CPR when it is not medically

appropriate/indicated.

“What do you know about CPR?”

“This decision seems very hard for you. I want to give you the

best medical care possible. Can you tell me more about your

decision?”

**NOTE:** If you will honor the request for CPR:

“I understand your desire for CPR, but I will need some direction if

you survive, since you will almost certainly be on a breathing machine

in an ICU. It is very likely that you will not be able to make decisions

for yourself. Who do you want to make decisions for you? Can you

give me some sense of how long we should continue life support if

you are not able to make decisions and there is no improvement in

your condition?”

** Expected death from chronic life-limiting diseases: advanced

metastatic cancer with poor and declining functional status, renal

failure on dialysis, multi-organ failure, advanced dementia, end-stage

liver or cardiac disease, etc.

**REFERENCES**

Abrahm JL. *A Physician’s Guide to Pain and Symptom Management in Cancer Patients*.

Baltimore, MD: Johns Hopkins University Press; 2000.

Cole S, Bird J. *The Medical Interview: The Three Function Approach*. 2nd ed. St. Louis, MO: Mosby; 2000.

Eener B. Empathy. In: Feldman MD, Christensen JF. *Behavioral Medicine in Primary Care: A Practical Guide.* Stamford, CT: Appleton & Lange; 1997:8-14.

Emanuel LL, von Gunten CF, Ferris FD,eds. The EPEC Curriculum. 1999. *The EPEC*

*Project:* https://www.bioethics.northwestern.edu/programs/epec/

Fast Facts and Concepts #4, 6, 11, 17, 19, 23, 24,26 ,29 ,38 ,55. Palliative Care Network of Wisconsin website. <https://www.mypcnow.org/fast-facts>.

Kleinman A, Eisenberg L, Good B. Culture, illness and care: Clinical lessons from

anthropologic and cross-cultural research. *Annals Int Med.* 1978;88:251-8.
