# Supplementary material for: Family Meeting Training Curriculum: A Multimedia Approach With Real-Time Experiential Learning for Residents
Source: MedEdPORTAL. 2020 Mar 6;16:10883. doi: 10.15766/mep_2374-8265.10883 (PMC7062545; doi:10.15766/mep_2374-8265.10883)
Supplement: Supplementary file 1 — A. Communication Basics.pptx B. Family Meeting E-Learning Project folder C. ICU Resident Orientation.pptx D. Family Meeting Resources Booklet.docx E. FMBS Tool.docx F. Global Self-Efficacy Survey.docx [file mep-16-10883-s001.zip › E. FMBS Tool.docx]

**Appendix E: Family meeting behavioral skills checklist for formative feedback** – used for identification of learning goals before and self-reflection / formative feedback after family meetings in the Medical Intensive Care unit

**A. Educational goals of the family meeting for the trainee:**

1. List the potential challenge(s) for you in this encounter

2. Identify at least 1-2 skills that you want to work on during this encounter

| **B. Behavioral skills checklist** | **Behavior Performed** | | |
| --- | --- | --- | --- |
|  | | | |
| **1. Prepare for family meeting** | **Yes** | **No** | **N/a** |
| Identified members of care team to be involved in meeting | 🞐 | 🞐 | 🞐 |
| Reviewed medical issues with care team | 🞐 | 🞐 | 🞐 |
| Discussed goals of family meeting with care team | 🞐 | 🞐 | 🞐 |
| Minimized distractions i.e. arranged a private location, turned off pagers/phones | 🞐 | 🞐 | 🞐 |
| Greeted pt./family members and asked for introductions | 🞐 | 🞐 | 🞐 |
| **2. Assess/Understand family and patient perception** | **Yes** | **No** | **N/a** |
| Expressed interest in patient as a person, prior to illness | 🞐 | 🞐 | 🞐 |
| Asked pt./family to share perspective on patient’s illness i.e. 3 levels of understanding | 🞐 | 🞐 | 🞐 |
| **3. Elicit pt./family preferences for communication** | **Yes** | **No** | **N/a** |
| Asked pt./family who the team should contact regarding patient condition | 🞐 | 🞐 | 🞐 |
| Asked pt./family about amount of detail that would be helpful re: clinical condition | 🞐 | 🞐 | 🞐 |
| Explored pt./family’s decision-making preferences | 🞐 | 🞐 | 🞐 |
| **4. Exchange/Share clinical information with pt./family** | **Yes** | **No** | **N/a** |
| Clearly stated the patient’s clinical condition with avoidance of medical jargon | 🞐 | 🞐 | 🞐 |
| Asked if the family understood the information conveyed i.e. ask-tell-ask | 🞐 | 🞐 | 🞐 |
| Gave “warning” of difficult news with time for preparation | 🞐 | 🞐 | 🞐 |
| Provided information in short “chunks” | 🞐 | 🞐 | 🞐 |
| Provided level of detail to match family’s desired level of detail | 🞐 | 🞐 | 🞐 |
| **5. Assessing/Attending to patient and family reactions** | **Yes** | **No** | **N/a** |
| Explored pt./family’s psychosocial and emotional concerns i.e. tell me more | 🞐 | 🞐 | 🞐 |
| Explored pt./family’s spiritual and cultural concerns | 🞐 | 🞐 | 🞐 |
| Acknowledged/accurately reflected family’s distress i.e. named emotion | 🞐 | 🞐 | 🞐 |
| Validated pt./family reaction i.e. empathetic statement | 🞐 | 🞐 | 🞐 |
| **6. Mange uncertainty** | **Yes** | **No** | **N/a** |
| Assed prognostic awareness i.e. current understanding, future hope, and worry | 🞐 | 🞐 | 🞐 |
| Identified uncertainty of patient’s clinical trajectory i.e. hope/worry technique | 🞐 | 🞐 | 🞐 |
| Promoted normative coping i.e. hope for best/prepare for worst, denial/acceptance | 🞐 | 🞐 | 🞐 |
| **7. Share decision-making** | **Yes** | **No** | **N/a** |
| Achieved common understanding of patient’s clinical condition | 🞐 | 🞐 | 🞐 |
| Focused discussion on patient values/goals prior to discussion of specific interventions | 🞐 | 🞐 | 🞐 |
| Discussed treatment options based on patient’s goals/values | 🞐 | 🞐 | 🞐 |
| Offered recommendations when in keeping with family’s decision –making process | 🞐 | 🞐 | 🞐 |
| **8. Summarize/plan** | **Yes** | **No** | **N/a** |
| Summarized discussion | 🞐 | 🞐 | 🞐 |
| Suggested next steps including future contact between family and care team | 🞐 | 🞐 | 🞐 |
| Prepared family for the unexpected | 🞐 | 🞐 | 🞐 |
| Provided necessary resources/contact information to help support family | 🞐 | 🞐 | 🞐 |
| **9. General approach** | **Yes** | **No** | **N/a** |
| Used reflective questioning | 🞐 | 🞐 | 🞐 |
| Invited pt./family questions throughout meeting | 🞐 | 🞐 | 🞐 |
| Listened without interruption | 🞐 | 🞐 | 🞐 |
| Allowed silence | 🞐 | 🞐 | 🞐 |
| Demonstrated non-verbal cues of empathy/engagement | 🞐 | 🞐 | 🞐 |
| **10. Documentation** | **Yes** | **No** | **N/a** |
| Documented date and time of meeting in the chart | 🞐 | 🞐 | 🞐 |
| Documented members present for meeting | 🞐 | 🞐 | 🞐 |
| Summarized content of meeting accurately in chart | 🞐 | 🞐 | 🞐 |
| Documented family understanding of patient condition | 🞐 | 🞐 | 🞐 |
| Documented decision-making and subsequent plan | 🞐 | 🞐 | 🞐 |
| Documented and coordinated care to meet family support needs | 🞐 | 🞐 | 🞐 |
| Documented challenges including resolution and/or ongoing issues | 🞐 | 🞐 | 🞐 |

**C. Reflection on communication skills during Family Meeting:**

1. Did you perform the skills identified above (Section A)?

🞐 Yes

🞐 No

2. What went smoothly for you during this encounter?

3. What was most challenging for you about this encounter?

4. What do you take away from this encounter (i.e. take-home point)?

5. What skill would you like to work on during the next family meeting?

Reprinted with permission of the American Thoracic Society. Copyright © 2018 American Thoracic Society.

McCallister et al/2015/Communication Skills training Curriculum for Pulmonary and Critical Care Fellows/Annals of the American Thoracic Society/Vol 12, no.4

*Annals of the American Thoracic Society* is an official journal of the American Thoracic Society.
