# Supplementary material for: Family Meeting Training Curriculum: A Multimedia Approach With Real-Time Experiential Learning for Residents
Source: MedEdPORTAL. 2020 Mar 6;16:10883. doi: 10.15766/mep_2374-8265.10883 (PMC7062545; doi:10.15766/mep_2374-8265.10883)
Supplement: Supplementary file 1 — A. Communication Basics.pptx B. Family Meeting E-Learning Project folder C. ICU Resident Orientation.pptx D. Family Meeting Resources Booklet.docx E. FMBS Tool.docx F. Global Self-Efficacy Survey.docx [file mep-16-10883-s001.zip › F. Global Self-Efficacy Survey.docx]

Appendix F – Resident Self-Assessment Survey

1) How many family conferences did you **observe** during your most recent MICU rotation? *(You were in the room, but did not participate other than small talk, etc.)*

Zero 1-3 4-6 7-10 >10

3) How many family conferences did you **participate in, but not as the primary facilitator,** during your most recent MICU rotation? *(You may have contributed some information, but you were not the “driver” of the meeting).*

Zero 1-3 4-6 7-10 >10

4) How many family conferences did you **serve as the primary facilitator for** during your most recent MICU rotation?

Zero 1-3 4-6 7-10 >10

5) When I facilitate a family meeting,

a) I effectively develop rapport with family members.

All of the time most of the time sometimes rarely never I have never facilitated a meeting

b) I ask open-ended questions effectively.

All of the time most of the time sometimes rarely never I have never facilitated a meeting

c) I elicit all of the family’s agenda items.

All of the time most of the time sometimes rarely never I have never facilitated a meeting

d) I listen for and respond to cues to the family’s ideas, concerns, and expectations.

All of the time most of the time sometimes rarely never I have never facilitated a meeting

e) I address feelings and emotions with the family.

All of the time most of the time sometimes rarely never I have never facilitated a conference

f) I check for understanding and agreement from the family when deciding on a plan of care.

All of the time most of the time sometimes rarely never I have never facilitated a conference

6) Overall, I would rate my skills at family meeting facilitation as:

Excellent Good Adequate Inadequate Poor Don’t know

7. Please list three things (or more) that you have learned about family meetings during your MICU rotation

1.

2.

3.

8. Rate the importance of the following elements of a family meeting from 1-5:

a. pre-meeting :

1- not important 2- somewhat important 3- average 4- very important 5- essential

b. meeting:

1- not important 2- somewhat important 3- average 4- very important 5- essential

c. post-meeting/debriefing:

1- not important 2- somewhat important 3- average 4- very important 5- essential

11 Compare your family meeting skills from the start of the block to the end.

Beginning: Rate yourself: 1- novice 2- beginner 3- average 4-proficient 5- expert

End: Rate yourself: 1- novice 2- beginner 3- average 4-proficient 5- expert

12 If feedback is important to you, can you identify impediments to getting feedback for how you facilitate family meetings?

13 What family meeting facilitation skills have you improved upon during your MICU rotation?

Some items adapted from:

Lang F, McCord R, Harvill L, Anderson DS. Communication assessment using the Common Ground Instrument: psychometric properties. Fam Med 2004; 36(3):189-98.
