# Supplementary figures and images for: Family Meeting Training Curriculum: A Multimedia Approach With Real-Time Experiential Learning for Residents
Source: MedEdPORTAL. 2020 Mar 6;16:10883. doi: 10.15766/mep_2374-8265.10883 (PMC7062545; doi:10.15766/mep_2374-8265.10883)

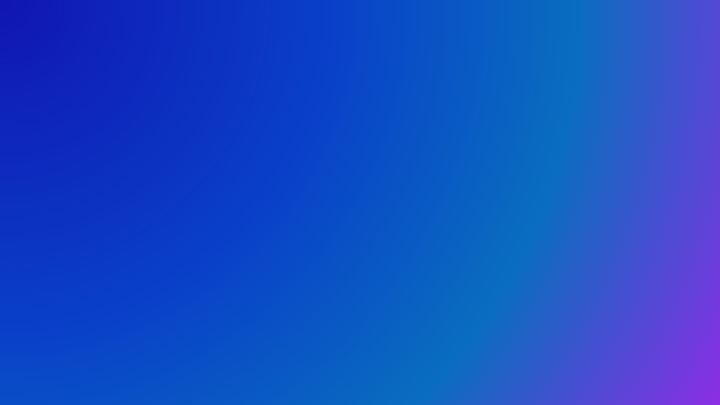

Supplement: Supplementary file 1 — A. Communication Basics.pptx B. Family Meeting E-Learning Project folder C. ICU Resident Orientation.pptx D. Family Meeting Resources Booklet.docx E. FMBS Tool.docx F. Global Self-Efficacy Survey.docx [file mep-16-10883-s001.zip › B. Family Meeting E-Learning Project folder/mobile/Slide6GNwsy36jIi.jpg]

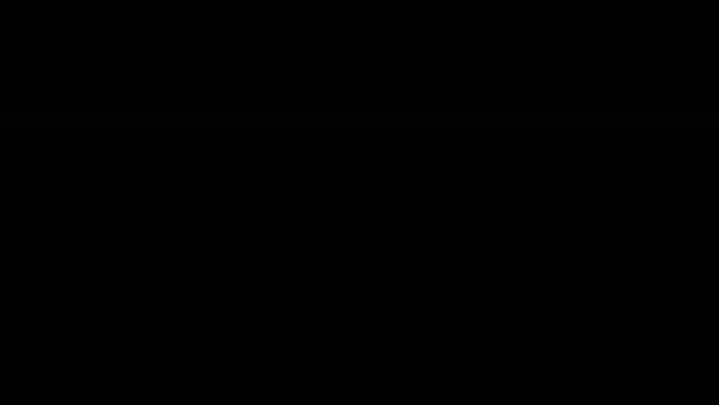

Supplement: Supplementary file 1 — A. Communication Basics.pptx B. Family Meeting E-Learning Project folder C. ICU Resident Orientation.pptx D. Family Meeting Resources Booklet.docx E. FMBS Tool.docx F. Global Self-Efficacy Survey.docx [file mep-16-10883-s001.zip › B. Family Meeting E-Learning Project folder/mobile/video_5mkWMYbMOxP_22_48_720x406.jpg]

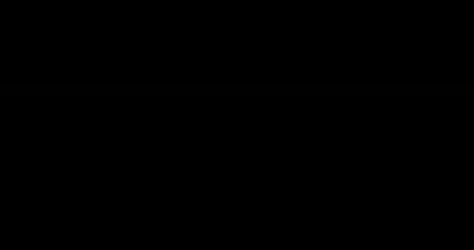

Supplement: Supplementary file 1 — A. Communication Basics.pptx B. Family Meeting E-Learning Project folder C. ICU Resident Orientation.pptx D. Family Meeting Resources Booklet.docx E. FMBS Tool.docx F. Global Self-Efficacy Survey.docx [file mep-16-10883-s001.zip › B. Family Meeting E-Learning Project folder/mobile/video_5oGNorpuHhN_22_48_630x332.jpg]

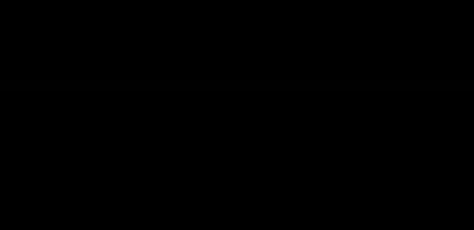

Supplement: Supplementary file 1 — A. Communication Basics.pptx B. Family Meeting E-Learning Project folder C. ICU Resident Orientation.pptx D. Family Meeting Resources Booklet.docx E. FMBS Tool.docx F. Global Self-Efficacy Survey.docx [file mep-16-10883-s001.zip › B. Family Meeting E-Learning Project folder/mobile/video_6bBJwiXemHd_22_48_662x322.jpg]

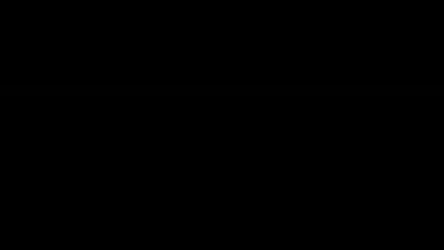

Supplement: Supplementary file 1 — A. Communication Basics.pptx B. Family Meeting E-Learning Project folder C. ICU Resident Orientation.pptx D. Family Meeting Resources Booklet.docx E. FMBS Tool.docx F. Global Self-Efficacy Survey.docx [file mep-16-10883-s001.zip › B. Family Meeting E-Learning Project folder/mobile/video_6BI73PvOwgo_22_48_578x326.jpg]

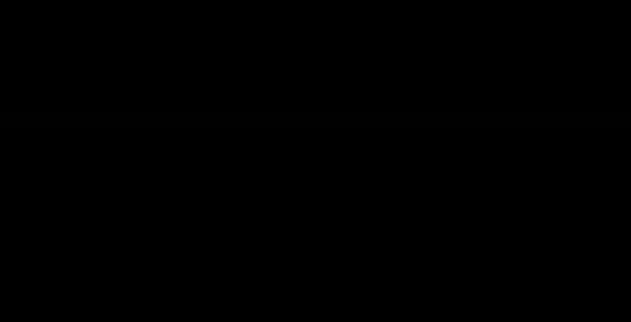

Supplement: Supplementary file 1 — A. Communication Basics.pptx B. Family Meeting E-Learning Project folder C. ICU Resident Orientation.pptx D. Family Meeting Resources Booklet.docx E. FMBS Tool.docx F. Global Self-Efficacy Survey.docx [file mep-16-10883-s001.zip › B. Family Meeting E-Learning Project folder/mobile/video_6DmllvncV2s_22_48_630x322.jpg]

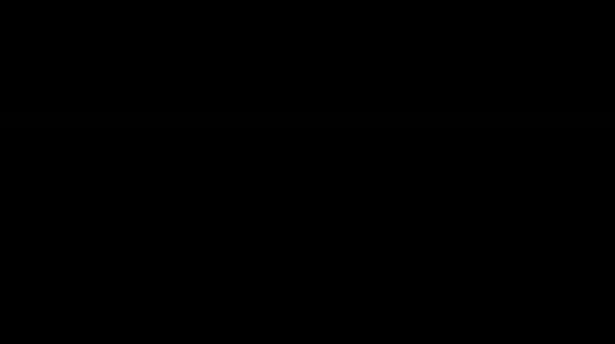

Supplement: Supplementary file 1 — A. Communication Basics.pptx B. Family Meeting E-Learning Project folder C. ICU Resident Orientation.pptx D. Family Meeting Resources Booklet.docx E. FMBS Tool.docx F. Global Self-Efficacy Survey.docx [file mep-16-10883-s001.zip › B. Family Meeting E-Learning Project folder/mobile/video_6ecdBkJzMcm_22_48_348x196.jpg]

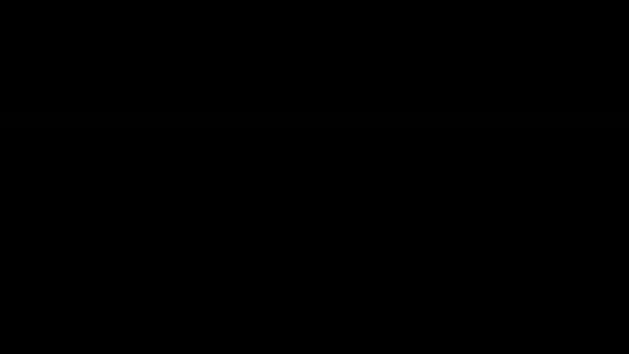

Supplement: Supplementary file 1 — A. Communication Basics.pptx B. Family Meeting E-Learning Project folder C. ICU Resident Orientation.pptx D. Family Meeting Resources Booklet.docx E. FMBS Tool.docx F. Global Self-Efficacy Survey.docx [file mep-16-10883-s001.zip › B. Family Meeting E-Learning Project folder/mobile/video_6F0m3ikfHEy_22_48_346x196.jpg]

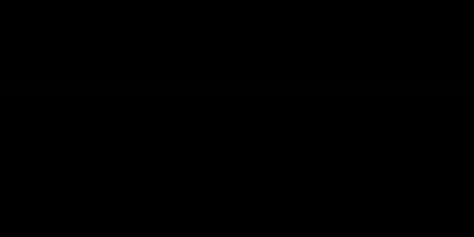

Supplement: Supplementary file 1 — A. Communication Basics.pptx B. Family Meeting E-Learning Project folder C. ICU Resident Orientation.pptx D. Family Meeting Resources Booklet.docx E. FMBS Tool.docx F. Global Self-Efficacy Survey.docx [file mep-16-10883-s001.zip › B. Family Meeting E-Learning Project folder/mobile/video_6HpzQcFZMt5_22_48_664x332.jpg]

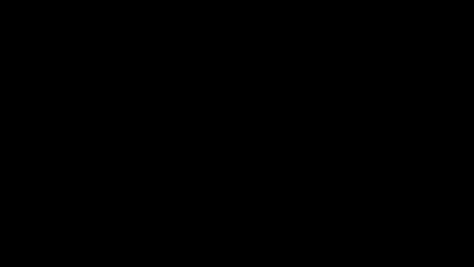

Supplement: Supplementary file 1 — A. Communication Basics.pptx B. Family Meeting E-Learning Project folder C. ICU Resident Orientation.pptx D. Family Meeting Resources Booklet.docx E. FMBS Tool.docx F. Global Self-Efficacy Survey.docx [file mep-16-10883-s001.zip › B. Family Meeting E-Learning Project folder/mobile/video_6HV1kXzf1Xw_22_48_588x332.jpg]

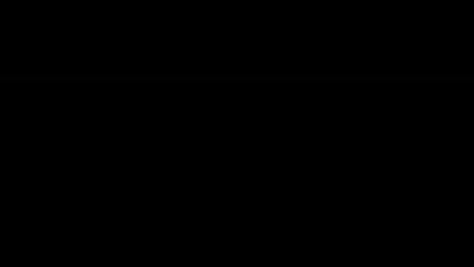

Supplement: Supplementary file 1 — A. Communication Basics.pptx B. Family Meeting E-Learning Project folder C. ICU Resident Orientation.pptx D. Family Meeting Resources Booklet.docx E. FMBS Tool.docx F. Global Self-Efficacy Survey.docx [file mep-16-10883-s001.zip › B. Family Meeting E-Learning Project folder/mobile/video_6N7c3eIhUDk_22_48_628x354.jpg]

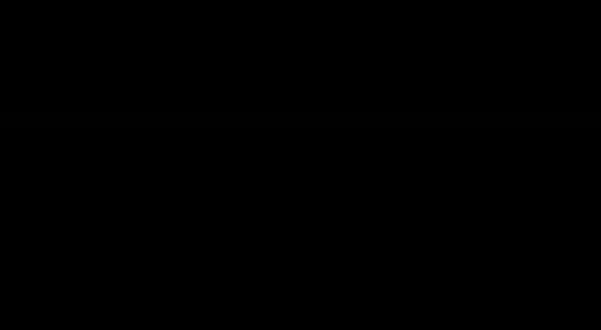

Supplement: Supplementary file 1 — A. Communication Basics.pptx B. Family Meeting E-Learning Project folder C. ICU Resident Orientation.pptx D. Family Meeting Resources Booklet.docx E. FMBS Tool.docx F. Global Self-Efficacy Survey.docx [file mep-16-10883-s001.zip › B. Family Meeting E-Learning Project folder/mobile/video_6XychCw337N_22_48_600x330.jpg]

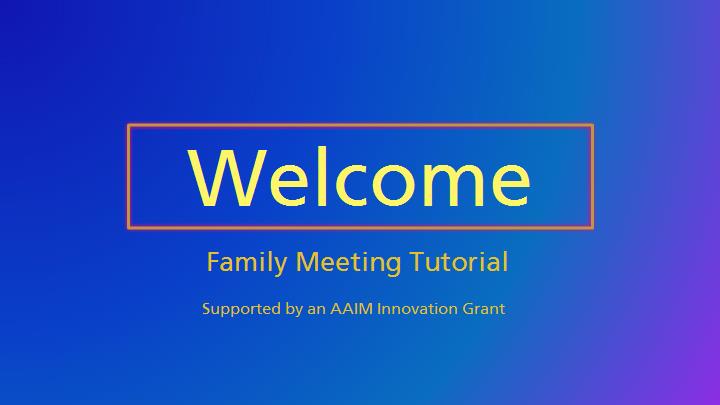

Supplement: Supplementary file 1 — A. Communication Basics.pptx B. Family Meeting E-Learning Project folder C. ICU Resident Orientation.pptx D. Family Meeting Resources Booklet.docx E. FMBS Tool.docx F. Global Self-Efficacy Survey.docx [file mep-16-10883-s001.zip › B. Family Meeting E-Learning Project folder/story_content/thumbnail.jpg]

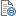

Supplement: Supplementary file 1 — A. Communication Basics.pptx B. Family Meeting E-Learning Project folder C. ICU Resident Orientation.pptx D. Family Meeting Resources Booklet.docx E. FMBS Tool.docx F. Global Self-Efficacy Survey.docx [file mep-16-10883-s001.zip › B. Family Meeting E-Learning Project folder/story_content/url.png]
